# Historical data for conservation: reconstructing range changes of Chinese pangolin (*Manis pentadactyla*) in eastern China (1970–2016)

**DOI:** 10.1098/rspb.2018.1084

**Published:** 2018-08-22

**Authors:** Li Yang, Minhao Chen, Daniel W. S. Challender, Carly Waterman, Chao Zhang, Zhaomin Huo, Hongwei Liu, Xiaofeng Luan

**Affiliations:** 1 School of Nature Conservation, Beijing Forestry University, No. 35 Tsinghua East Road Haidian District, Beijing 100083, People's Republic of China; 2 IUCN SSC Pangolin Specialist Group, Zoological Society of London, Regent's Park, London NW1 4RY, UK; 3 School of Life Sciences, Sun Yat-sen University, No. 135, Xingang Xi Road, Guangzhou, Guangdong 510275, People's Republic of China; 4 Department of Life Sciences, Imperial College, Silwood Park Campus, London, UK

**Keywords:** Chinese pangolin, conservation, eastern China, historical range, *Manis pentadactyla*

## Abstract

The Chinese pangolin (*Manis pentadactyla*) has long suffered from intense exploitation driven by consumer demand for medicinal use and food. Effective conservation management is hampered by insufficient data on pangolin status and distribution. We integrated ecological niche modelling with long-term ecological records at the local scale (e.g. from local historical documents, grey and published literature and interviews) to estimate the magnitude of potential distribution change of the Chinese pangolin in eastern China (Fujian, Jiangxi and Zhejiang provinces) over time. Our results suggest that the range of the species decreased by 52.20% between the 1970s and early 2000s and that the population is now mainly confined to the Wuyi Mountains. This reduction in potential distribution range is attributable to anthropogenic pressures. According to our conservation prioritization analysis, the priority conservation area for the Chinese pangolin in eastern China is 51 268.4 km^2^, 5.62% of which is covered by nature reserves. There are 18 nature reserves and 46 prefectures which are priority areas for conservation in China. The priority-level nature reserves and prefectures in eastern China are mainly located in the centre of the Wuyi Mountains, and areas declared important tend to be around the Wuyi Mountains. We propose several actions to improve the conservation status of this species: establish or enlarge nature reserves, ensure local governments at the prefecture level prioritize conservation management and encourage local communities to participate in pangolin conservation.

## 1. Introduction

Pangolins (Pholidota: Manidae), African and Asian scaly mammals comprising eight species, are considered to be the most heavily trafficked wild mammals in the world [1–3]. Asian pangolins, especially the Chinese pangolin (*Manis pentadactyla*) and Sunda pangolin (*Manis javanica*), are poached and trafficked in large volumes for traditional medicines and food [1,2,4]. Owing to a long history of exploitation driven by growing demand for their parts, Chinese pangolin populations have dramatically decreased, and the species is listed as ‘Critically Endangered’ on The International Union for Conservation of Nature (IUCN) Red List of Threatened Species [5,6]. It is also listed in Appendix I of the Convention on International Trade in Endangered Species of Wild Fauna and Flora (CITES), banning international commercial trade in wild Chinese pangolins and their parts or derivatives (CITES 2017, https://cites.org/eng/app/appendices.php). Within the last decade, research on pangolins has focused primarily on levels of illegal trade, and on captive breeding and behaviour, and there has been little focus on population studies for these species [4,6–9]. Moreover, few studies have focused on the distribution and population of Chinese pangolins on a large spatial scale [10,11]. While these studies suggest that populations have decreased significantly, they provide insufficient data for designing conservation initiatives. There is, therefore, an increasing and unmet need to assess basic distribution data to support conservation efforts [2].

Previous research has highlighted that collating local-scale data (e.g. from markets or sighting records at local scales) can inform accurate estimates of species distribution [3,6,12,13]. Compared with local history documents, these data tend to provide information on the status of species distribution but are not sufficient to provide changes in species distribution over time. To overcome this barrier, analysing long-term ecological records from local historical documents can potentially supplement understanding of species distribution and status, e.g. by detecting major shifts in records of occurrence with a historical perspective [14–18]. The compilation of local historical documents is systematized across most of China and has been maintained at a reasonably high spatial resolution (mostly at the prefecture level) since 1950 [19]. These documents often include objective information on species at a local level, including distribution, population estimates and local trade [14,20]. We recognized that combining this local information with long-term ecological records at local scales offers an opportunity to understand the ecological and biogeographical characteristics of an endangered species through time.

In practice, primary data at local scales is often incomplete and spatially biased, and these problems can be reduced by ecological niche modelling [15,20,21]. Discrepancies among different species distribution models (SDMs) can be large, making it difficult to choose an appropriate model [22,23]. In this context, ensemble forecasting approaches may be an appropriate choice [24]. BIOMOD is considered a suitable platform for ensemble forecasting of species distributions [24,25]. Combining multiple data resources at local scales with ecological niche modelling can facilitate sensitive and comprehensive evaluation of a species by revealing changes that occur over decades.

Pangolin populations in eastern China, including Jiangxi, Fujian and Zhejiang Provinces, have long suffered from exploitation and illegal trade. It is estimated that populations in Fujian province have declined by up to 80% since the 1960s [10,26]. Yongxiu (Jiangxi Province) populations declined by 90% between 1964 and 1982 [27]. Sporadic data in Zhejiang Province also depict a dramatic decrease [28]. Population declines typically represent extended processes of reductions in species range and numbers, which may take decades or even longer to run their course [18,29–31]. Inconsistent data can only present a summary of the population decline and are insufficient to estimate the magnitude of change and meet conservation needs. We integrated ecological niche modelling with long-term ecological records to estimate the magnitude of change in Chinese pangolin distribution in eastern China over time. To achieve this, we (i) collected and combined local information with local historical documents; (ii) reconstructed climate data to represent environmental variables; (iii) estimated the ecological niche changes and potential habitats for each period; and (iv) evaluated changes in the pangolin distribution range and their possible impact factors.

## 2. Methods

### (a) Study area

The study area is in eastern China [20] and comprises three provinces, Fujian Province, Jiangxi Province and Zhejiang Province (23°34'–31°11' N, 113°34′–122°57′ E). This area includes two mountain ranges and a plain and is characterized by hills, particularly the Zhe-min Hills. The region has a subtropical monsoon climate with high rainfall (1486–2150 mm yr^−1^) year-round [32]. The habitat is largely composed of broadleaf forest and mixed broadleaf–conifer forest, which supports a number of forest-dependent mammals including the Chinese pangolin.

**Figure 1.**
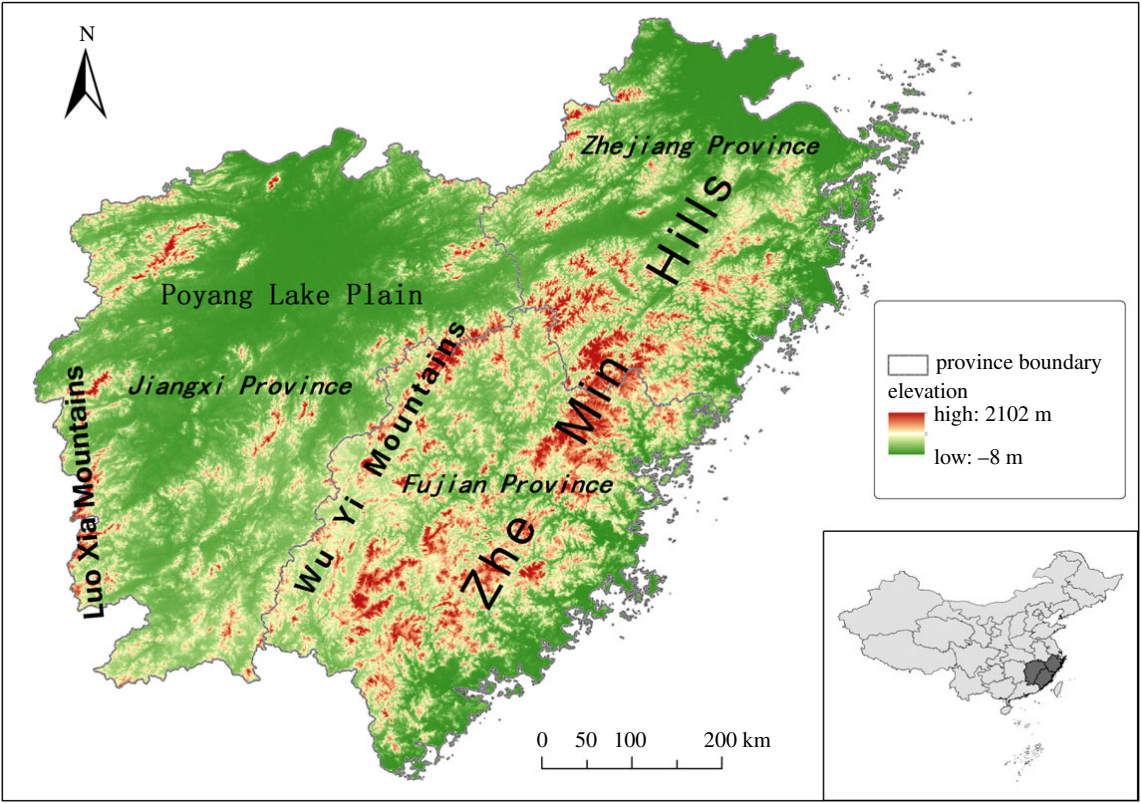
Study area—Eastern China (include Zhejiang Province, Fujian Province and Jiangxi Province). (Online version in colour.)

In this study, we obtained the boundaries of Eastern China from the Thematic Database for Human–Earth System (http://www.data.ac.cn/index.asp). The area covers 512 684 km^2^ with an elevation range from −8 to 2102 m. The ecological data were obtained in a raster structure with a cell size of 1 km^2^ (figure 1).

### (b) Data

Following the data collection process described in our previous research [14–17,20], we collected pangolin data from five sources: local historical documents, fauna records, nature reserve scientific surveys, published scientific research (including articles and specimen records) and news articles. We also conducted semi-structured interviews with local people to collect data for occurrences after the year 2000 [12,33,34]. Conflicting records with unsubstantiated metadata, such as those lacking relevant or detailed descriptions, were excluded from the analysis. We recorded locality information from all of the extracted records on Google Earth (see details in electronic supplementary material, appendix SI). To eliminate potential bias caused by clustered occurrences, we removed duplicate records according to the home range of the Chinese pangolin (due to a lack of reliable information on the size of Chinese pangolin home ranges, we used home range of the Sunda pangolin, *Manis javanica* for analysis, which is 6.97 ha [12], see details in electronic supplementary material, appendix SI). Ultimately, we obtained 714 occurrence data points in the 1970s (1970–1979), 561 in the 1980s (1980–1989), 322 in the 1990s (1990–1999) and 117 in the 2000s (2000–2016).

Climate data (mean annual temperature, MAT; mean annual precipitation; the number of frost-free days, NFFD; Hargreaves reference evaporation, Eref) were downloaded from Climate AP v. 2.03 (http://climateap.net/; [35]); topography data (elevation and aspect) were obtained from the SRTM 90 m Digital Elevation Database (http://srtm.csi.cgiar.org/); forested and urban land were extracted from land cover data (dataset provided by the Data Center for Resources and Environmental Sciences, Chinese Academy of Sciences (RESDC; http://www.resdc.cn)). A total of eight variables were obtained for further research (see details in electronic supplementary material, appendix SII).

### (c) Niche changes over time

Measuring the contribution of each period to the total niche of the species (i.e. the niche as measured considering all available points) can be helpful to elucidate the reasons underlying range changes [36,37]. The niche here tends to be a realized niche, for SDMs are in fact based on the empirical relationships between observed species distributions and environmental variables [38]. Based on the methods of Broennimann *et al*. [36] and Maiorano *et al*. [37], we measured niche conservatism in a gridded and smoothed principal components analysis (PCA) environmental space (PCA-env), accounting for environmental availability (i.e. the values of the climate variables over the study area for each time frame) (see details in electronic supplementary material, appendix SII).

### (d) Species distribution modelling

The collection of historical records was limited by the capacity of transportation at that time, recording more records in traffic-prone areas than traffic-lagging areas [39–41]. Therefore, we obtained road data from RESDC (http://www.resdc.cn). We created 10 000 random points from the buffer zone within 15 km of the road dataset (we only selected main roads that had existed for more than 30 years).

Collinearity problems can lead to bias, so it is necessary to reduce overfitting for modelling. Following the suggestions of Dormann *et al*. [42], we used pairwise diagnostic tools (VIF, variance inflation factor) in this study. This analysis was calculated using the ‘BiodiversityR’ package in R (threshold = 10). For each period, eight variables were selected for modelling, except for the 1980s, when MAT was excluded; see details in electronic supplementary material, appendix SII.

We estimated the potential distribution of the Chinese pangolin in eastern China over time using BIOMOD2. We used eight modelling techniques implemented in the BIOMOD2 R package (v. 3.3–7) [24,43]: generalized additive modelling, generalized boosting modelling (GBM), generalized linear modelling, artificial neural network (ANN), flexible discriminant analysis, multivariate adaptive regression splines, random forest, and maximum entropy (MAXENT. Phillips). All techniques were applied to the presence data for each period and to the pseudo-absences for the models developed in BIOMOD2. For each period, each SDM was evaluated by measuring the true skill statistic (TSS) [44] with 10 evaluation runs. For the TSS, 80 modelling evaluation results were obtained, and the average TSS was set as the threshold for building the ensemble models. Then, the potential distribution was calculated from an ensemble set of models or predictions. To reduce the uncertainty in our research, we repeated the models 10 times, yielding a total of 320 SDMs from the historical periods (the 1970s, 1980s, 1990s and 2000s). To transform the models from environmental suitability into the presence–absence distributions, we used the threshold (*P*, cut-off) calculated by BIOMOD2. From this, all outputs were divided into two groups: outputs above the threshold (>*P*) were grouped as ‘present’, whereas all other values (<*P*) were considered ‘absent’. Finally, the potential distribution through time was obtained.

The spatial analyses were conducted in ArcGIS (v. 10.2; ESRI, Inc., Redlands, CA, USA), Excel 2013 (Microsoft Corp., Redmond, WA, USA), R v. 3.4.2 (2017) and SPSS for Windows (v. 20.0; SPSS, Inc., Chicago, IL, USA).

### (e) Conservation prioritization analysis

Conservation prioritization should address the current status of species. We used the core-area zonation algorithm in Zonation 4.00rc1 [45] to prioritize the landscape for conservation protection. Zonation produces a complementarity-based and balanced ranking of conservation priority over the entire landscape. The priority ranking is produced by iteratively removing the grid cell or planning unit that leads, according to the cell's contribution to the population or distribution of the species, to the smallest aggregate loss of conservation value. For each iteration, the algorithm seeks to maintain core areas to create a ranking by importance for maintaining the species [46].

We ran the core-area algorithm with the probability of occurrence models (2000s output) as inputs. Because human influence and vegetation can be critical factors to the Chinese pangolin, we selected urban land, forestry and vegetation cover in the 2000s as the condition layers (see details in electronic supplementary material, appendix SII). Normally, the cost layer is associated with the cost of protecting the particular site. Here, we assumed that the time of animal disappearance was associated with the cost: the longer it is absent, the harder it is to recover the population. We overlaid the SDMs results for each period and converted to the cost layer; values ranged from 0 to 1, with ‘0’ indicating exit in the 2000s, and ‘1’ absent since the 1970s (see details in electronic supplementary material, appendix SIII). We reclassified the final output into four groups: greater than 0.95, Mandatory Reserve; 0.95–0.9, Negotiated Reserve; 0.8–0.89, Partial Reserve; less than 0.8, Other area. In addition, data for 39 nature reserves in the study area were collected to reveal the relationship between the conservation priority area and the existing conservation network (see details in electronic supplementary material, appendix SIII). As the basic unit for conservation management, local prefecture-level governments in the study area were identified and assigned to conservation management units.

## 3. Results

### (a) Niche changes through time

**Figure 2.**
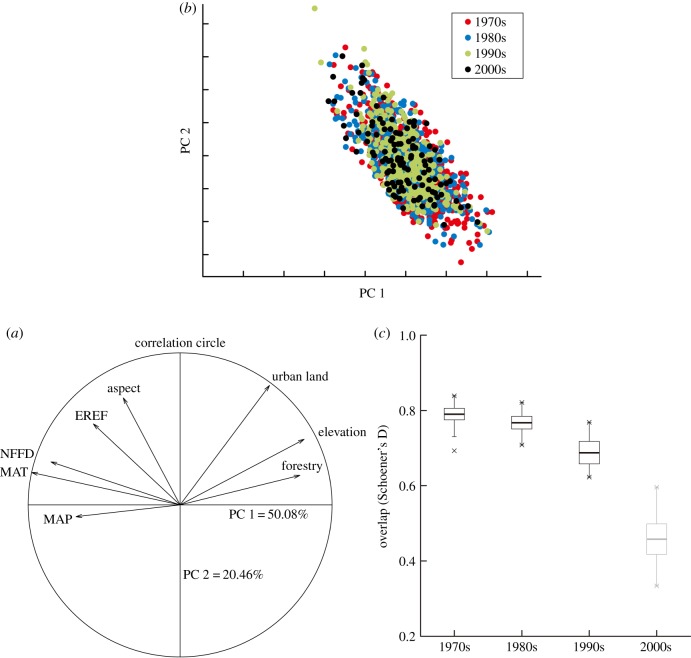
(*a*) Species niches projected in the environmental space defined by PCA calibrated on all environmental variables for all periods (see text for more details). PC1 and PC2 represent the first two principal components, explaining 70.54% of the total variability. The correlation circle shows the variance contribution of axes 1 (PC1) and 2 (PC2). (*b*) Dots represent species distribution in the environmental space; and different colours represent different time periods. (*c*) Comparison between partial niche and total niches for each time period. Box plots represent Schoener's D values measured considering 100 random subsamples of the total niche with a sample size equivalent to the occurrence data available for each period. The bold lines indicate the mean values, X's indicate extreme values, and light grey boxes indicate non-significant relationships. (Online version in colour.)

The first two axes of the PCA calibrated over all periods and considering all variables accounted for 70.54% of the total variability in the variables (50.08% of variability along the first axis, 20.46% along the second axis; figure 2*a*), with two main gradients, one corresponding to climate and human influence, and the other to human influence.

Contraction of the Chinese pangolin's range (figure 2*b*) over time occurred along the human influence gradient rather than the climate gradient. In the earlier time frames, Chinese pangolins had a wild niche with intermediate climate conditions and human influence, and contracting into lower human influence through time to the present.

The overlap between the partial and total niches changed through time (figure 2*c*) and was lower in the 2000s than in the 1970s (figure 2*c*). Niche similarity was statistically significant for all comparisons, with the exception of the 2000s (*p*-value = 0.06 > 0.05).

### (b) Model performance

Based on the available records for the Chinese pangolin at different periods in our study, the records ranged from 117 to 714 (table 1). The average TSS value for the consensus models was 0.68 (table 1).

**Table 1.** Estimates of range size and accuracy of species distribution models in eastern China in different time periods. Occurrence point data were collected from six sources and verified (see Methods—Data). Model accuracy was measured by the TSS (see details in appendix SII). For the relative importance of variables to the Chinese pangolin, only the most important variable is shown (see details in electronic supplementary material, appendix SII). The ranges were determined as the total number of related pixels.

period	occurrences	TSS	relative importance	range (km^2^)	elevation (m)
1970s	714	0.593	elevation	155 929	631
1980s	561	0.657	elevation	104 093	699
1990s	322	0.712	elevation	92 271	716
2000s	117	0.764	elevation	74 537	716

On average, most internal model evaluation provided fair or good results [47], with most TSS values greater than 0.40 (see details in electronic supplementary material, appendix SII), excepting ANN in the 1970s (TSS = 0.35). On average, GBM had the highest TSS values, and ANN had the lowest. Because the average TSS was set as the threshold for building the ensemble models, approximately 50% of the models were excluded for each period (leaving the 50% of models with the highest TSS values). In general, model accuracy increased with time frames closer to the present. The period from 2000 to 2016 had the highest TSS value among all periods, and the 1970s had the lowest value.

Elevation was by far the most important variable across all periods, followed by MAT and NFFD.

### (c) Range change through time

**Figure 3.**
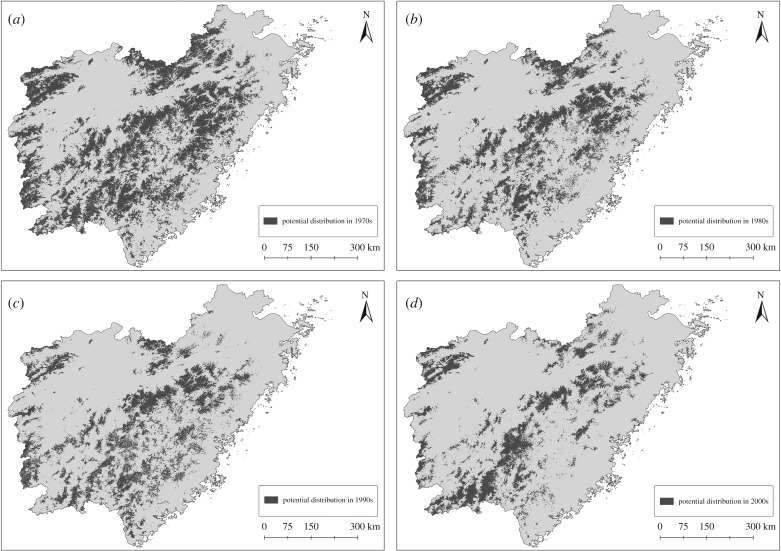
Potential distribution of the Chinese pangolin (*Manis pentadactyla*) in different time periods. Notes: Maps (*a*–*d*) represent potential distributions of the Chinese pangolin. 1970s = 1970–1979; 1980s = 1980–1989; 1990s = 1990–1999 and 2000s = 2000–2016.

Our results show that Chinese pangolins were once widely distributed in eastern China, especially in the mountainous area (figure 3). In the 1970s, the area of potential distribution reached 30.41% of eastern China. Compared with the area of potential distribution in 1970s, the situation worsened after that, with a reduction of 33.24% in the 1980s, 40.32% in the 1990s and 52.20% in the 2000s. The average rates of decrease since the 1970s have been 21.27% per decade (33.24% between the 1970s and the 1980s, 11.36% between the 1980s and the 1990s, 19.22% between the 1990s and the 2000s). The average elevation has increased for each period since the 1970s (table 1). Eventually, the distribution became concentrated in the mountainous area in 2000–2016 (especially in the Wuyi Mountains).

### (d) Conservation prioritization

**Figure 4.**
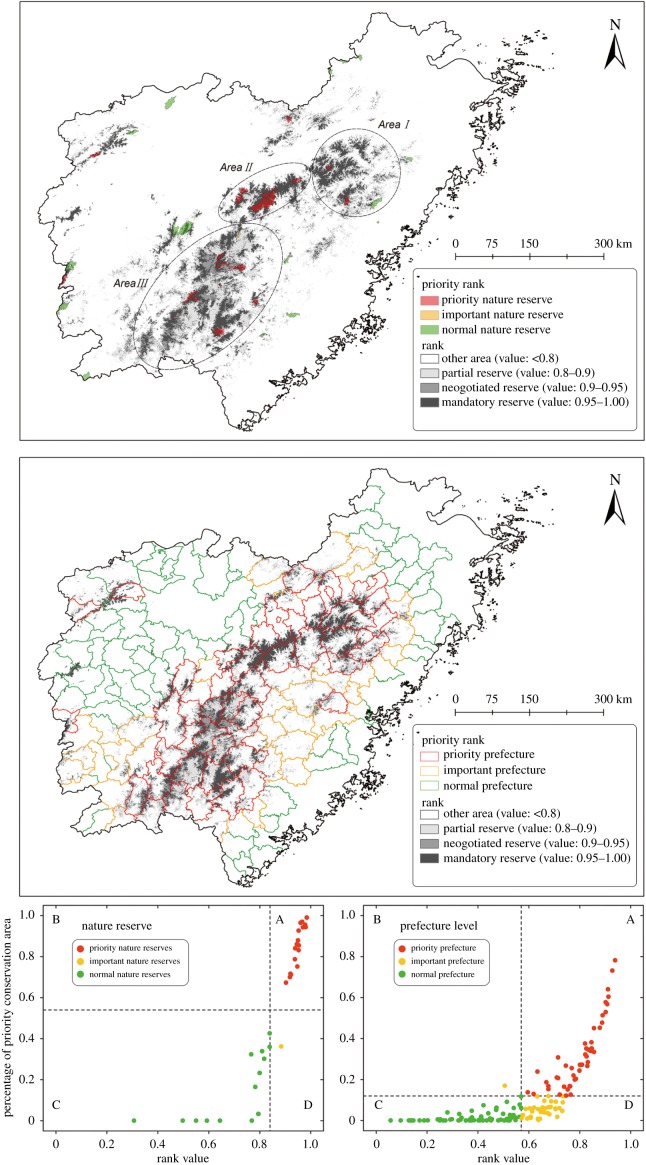
(1) Results from the zonation conservation ranking of the landscape with the cost layer. (2) The priority nature reserves and prefectures according to zonation conservation ranking. Red dots indicate priority level, yellow dots important level and green dots indicate normal level. Bottom left: the dashed-line *y*-axis represents the average percentage of priority conservation areas. Bottom right: the dashed-line *y*-axis indicates the average rank for the 33 nature reserves or 151 prefectures. (Online version in colour.)

Understanding the influences and dynamics of range changes at longer temporal scales is important for conservation and environmental management practices. According to the core-area zonation algorithm result and the rank value from 0 to 1, with 1 representing the highest priorities (see details in electronic supplementary material, appendix SIII), three conservation priority ranks were identified: the top 5% (25 634.2 km^2^) was considered Mandatory Reserves; the next 5% (25 634.2 km^2^) was Negotiated Reserves and 10% (51 268.4 km^2^) was Partial Reserves (figure 4). In addition, we suggest that the top 10% of the ranked areas be considered the priority conservation areas. These areas are primarily located in the Wuyi Mountains (figure 3).

The mandatory reserves and negotiated reserves were considered the priority conservation areas. The nature reserves of the potential distribution in the 2000s were excluded from further analysis (six nature reserves were excluded for their data shortage). Calculated over 33 nature reserves in eastern China, the rank value on average was 0.84 ± 0.16, completely covering 5.62% of the priority conservation area, and the percentage for each nature reserve was 0.54 ± 0.36 (only 81.81% of nature reserves had priority conservation areas). With respect to the three nature reserve levels, 18, 1 and 14 areas were defined as priority, important and normal reserves, respectively. The priority-level nature reserves are mainly located in the centre of the Wuyi Mountains, and those identified as important nature reserves tend to be around the Wuyi Mountains. In China, local government can be the main participant in biological conservation, so we also carry out conservation priority analysis at prefecture level. Some prefectures with low conservation values were excluded based on the potential distribution in the 2000s (34.91% were excluded; see details in electronic supplementary material, appendix SIII). We also reclassified the local governments at the prefecture level into three levels following the rule for nature reserves in China. Priority, important and normal designations were applied to 46, 36 and 69 local governments, respectively. Most priority prefectures are located in the Wuyi Mountains, and those designated as important are restricted to the areas around the Wuyi Mountains.

## 4. Discussion

By collating local-scale data (five data sources) over a long timescale combined with semi-structured interviews, we provided the first regional estimates of changes in the Chinese pangolin distribution range in eastern China through time. Our investigation also provides an important new resource in combining multiple data sources with an SDM to reconstruct the temporal and spatial dynamics of an endangered species' distribution changes. Our results suggest that the outlook for the pangolin may still be bleak, and the conservation priority areas require immediate conservation action.

### (a) Niche change and threats

Our results demonstrate that the realized niche of the Chinese pangolin considered in this study may have changed significantly since the 1970s. In our research, the partial climatic niches showed a significant overlap with the total niche, with maximum values of average Schoener's D near 0.8 and with values in three periods above 0.5 (figure 2); no individual time frame provided a complete agreement with the total niche. Our results also identify that niche change only occurred over several decades. Although the microclimate may remain stable in Chinese pangolin burrows, their habitat and food sources may still be affected by climate change [48,49]. This process of change in species may take a long time to run its course [30]. We suggest that niche change of the pangolin may not be able to be presented in such short timescale if climate change is the only one factor behind this process. Therefore, we conducted a simple test to determine whether climate change might have caused the decrease in range. We first assumed that human influence has stabilized since the 1970s or the 1980s. Then, we integrated ecological niche modelling with two assumption datasets (I: the climate layers in the 2000s+the human influence layers in the 1970s+the occurrence points in the 1970s and II: the climate layers in the 2000s+the human influence layers in the 1980s + the occurrence point in the 1980s) to estimate the range of the Chinese pangolin in the 2000s (see details in electronic supplementary material, appendix SIV). Ultimately, the results showed that the potential distribution under these two assumptions was higher in the 2000s than the ‘true’ result; i.e. these two assumptions resulted in an increasing trend compared with the 1970s or the 1980s. This test provides evidence that climate conditions may be a neutral factor or may be positive in some cases. Therefore, we conclude that niche compression has been mainly driven by human influence since the 1970s.

Although the microclimate of Chinese pangolin caves may remain stable, their habitats and food sources may still be affected by climate change [48,49]. In a study of ant invasions under climate change, ants eaten by pangolins increased in distribution in Southeast Asia as a result of climate change [50], consistent with our study's assumptions. Pangolin's passive defence strategy in dealing with human hunting has provided convenience for human hunting. This also clarified the impact of climate change and human influence on pangolins, human impact is more serious than expected.

In this case, the potential distribution can be captured by our approach due to several reasons: (i) historical records already contained potential information about human influence [15–17,20]; (ii) human influence layers for different periods were included for ensemble modelling; and (iii) quality historical climate data were obtained for each period [35].

Our results also show that the most important variable across all time periods was elevation, which was associated with human influence; the potential range has contracted since the 1970s, and the elevation of the range has increased through time. This is likely because low-altitude areas have developed transportation, so it is easy to transport captured pangolins to other provinces for consumption, and community development is also expanding from low to high altitudes. Consistent with the previous research, overexploitation due to consumer demand, rising prices and growing relative poverty is the most critical threat to the Chinese pangolin [1,51]. Retail prices for pangolin derivatives in China have been increasing over time [52,53]. The situation may worsen in the near future if consumption and demand for pangolin derivatives continues [1,3,6]. However, environmental protection programmes in China may have positive impacts on pangolin populations: the rate of decrease has been dropping since several environmental protection programmes were launched in the late 1980s, such as the PRC Law on the Protection of Wildlife, the Natural Forests Protection Program and establishing nature reserves [15,16,20].

### (b) Implications for conservation

Conservation actions should be applied on different scales. Based on conservation recommendations for pangolins according to the IUCN SSC Pangolin Specialist Group [2], we suggest that such actions be addressed on two scales in eastern China: the regional scale for three areas (figure 4) and the local scale for different areas—nature reserves and areas outside nature reserves.

(A) At the regional scale:
  (1) Field investigations should be initiated in all three areas to determine the status of populations, especially Area I, because most of the priority conservation areas are outside nature reserves and the patches are also concentrated.
  (2) Area II should also be prioritized for investigating the status of populations and subsequently, ecological monitoring systems should be established and populations monitored over time. Most Area II nature reserves are priority nature reserves.
  (3) The communities in mountain area are key to combating illegal wildlife trade (IWT). Their proximity to and knowledge of wildlife can help prevent IWT (e.g. illegal dealers purchasing from local villagers). Collaborative management is difficult to apply, because such communities are diverse and dispersive. There is an increasing need for applying law education and enforcement, supplemented by support for changes in livelihoods across these areas (cross prefectures, even provinces) [54]. Therefore, a union including priority nature reserves and priority prefectures for conservation management should be considered. Here we suggest that a pangolin national park covering three priority areas may be an effective measure. Then, an environmental management system including enforcement and community management across the three provinces can improve conservation effectiveness.
(B) At the local scale:
  (1) Local governments at the prefecture level can be the basic unit for conservation management. Field investigations to support conservation planning or basic data collection should be undertaken in the prefectures designated as priorities.
  (2) Illegal pangolin sales for medicine and food are still presented in rural areas. Strengthening the supervision of TCM pharmacies and catering in rural areas in Areas I–III is urgently needed.
  (3) In addition, Chinese pangolin products have been considered traditional Chinese medicine for several thousand years [55]. Pangolin conservation may not be only a simple conservation issue, but it is also a social issue which needs multidisciplinary integration.

## 5. Conclusion

Conservationists often focus on the current status of species but tend to overlook the historical context of range changes. We demonstrate that reconstructing range changes using long-term ecological data can reveal the processes and threats underlying such changes, which may not be detected by short-term research.

Such methods or datasets, for the species with unique characteristic or high economic/ecological value, can be generalized to other endangered species for maximizing profits. For further research, we suggest that: (i) conservation researchers and practitioners consider multiple data sources (not only the gazetteers, but also other data sources considered as local historical records around the world [20]) as complementary to reducing data shortages and enhancing overall understanding of species to inform conservation action; (ii) historical context can provide valuable information for endangered species assessments (e.g. improve our knowledge for IUCN Red List or CITES); and (iii) researchers and practitioners consider the historical background of endangered species for conservation planning.

## Supplementary Material

Appendix I Data collection

## Supplementary Material

Appendix II Environmental variables, niche analysis and model performance

## Supplementary Material

Appendix III Conservation Prioritization

## Supplementary Material

Appendix IV Influence from climate change

## Data accessibility

The datasets supporting this article have been uploaded as part of the supplementary material, see detail in electronic supplementary material, appendix SI Data collection. Climate data and geographical data are available from the Dryad Digital Repository at http://dx.doi.org/10.5061/dryad.db430s1 [56].
