# Supplementary material for: Historical data for conservation: reconstructing range changes of Chinese pangolin (Manis pentadactyla) in eastern China (1970–2016)
Source: Proc Biol Sci. 2018 Aug 22;285(1885):20181084. doi: 10.1098/rspb.2018.1084 (PMC6125891; doi:10.1098/rspb.2018.1084)
Supplement: Appendix I Data collection [file rspb20181084supp1.docx]

*Historical data for conservation: reconstructing range changes of Chinese pangolin (Manis pentadactyla) in eastern China (1970-2016)*

Li Yang, Minhao Chen, Daniel W.S. Challender, Carly Waterman, Chao Zhang, Zhaomin Huo, Hongwei Liu, Xiaofeng Luan

**Appendix I Data collection**

**A. Data collection rule**

1) Fauna records

The data from fauna records came from the specimens. The collection location can be obtained from the specimen label (which includes a place name or longitude and latitude). Four fauna records were obtained in total. We confirmed the location of each specimen with a historical remote sensing map (Google Earth) according to the species’ ecological requirements (dense vegetation and low human influence) [1-8].

2) Nature reserve scientific surveys

The records from nature reserve scientific surveys can be specimen records, small area name at the country level or the specific localities. A total of 14 records were obtained. We confirmed the location of each record with a historical remote sensing map (Google Earth) according to the species’ ecological requirements (dense vegetation and low human influence) [1-8].

3) Scientific research and news

Such resources record specific localities. 21 records were obtained. We confirmed the location of each record with a historical remote sensing map (Google Earth) according to the species’ ecological requirements (dense vegetation and low human influence) [1-8].

4) Local historical documents

a. Introduction

Local historical documents, also called local gazetteers or difangzhi (“地方志”), typically recorded considerable local environmental data, including wild animal records, as well as economic, political and demographic information. The compilation of local historical documents is systematized and provides dated geographical coverage across most of China at a reasonably high spatial resolution (mostly at the prefecture level) since 1950. These documents have been used to reconstruct numerous aspects of China’s environmental history and its relationship to past changes in Chinese society and economy. Recent research has demonstrated that these documents have potential value to biological conservation [9-14].

b. Species

Pangolins, also called “穿山甲” and “鳞鲤”, are considered to be an economically important species (meat for food, and scales for traditional medicine) by hunters and poachers. Pangolins also have special characteristics making it hard to confuse them with other species in eastern China. According to previous research, there is only one pangolin species (Chinese pangolin, *Manis pentadactyla*) living in eastern China [1, 3, 15]. Due to being an economically and culturally important species, pangolins are recorded regularly in local historical documents. The records from local historical documents provide a geographical coverage across most of eastern China.

c. Data resource

The information in local historical documents comes from several sources: 1) interview surveys with local people (e.g., hunters); 2) market records; 3) scientific investigations in forestry farms or nature reserves; 4) law enforcement records; 5) local medicine records. Due to the social development and transportation network in China before 2000, all the records can be considered as local records.

d. Time scale

Most gazetteer records do not record specific localities where pangolins occurred, but instead report their presence at the county level [16]. While many county-level boundaries have changed since the 1950s, the forestry farm and most country-level boundaries tend to have been stable since the 1970s. Therefore, our research addressed on the time scale from 1970 to 2015.

e. Occurrence points

Some gazetteer records describe pangolin presence at small place (e.g., they might record a local hill name or timber area) with the record time (usually year). We recorded the locality information from the extracted records on Google Earth.

Some gazetteers report pangolin presence at the county level (with a brief range, e.g. ‘northern mountain area’). In this case, we assumed that the records come from the scientific investigation for a forestry farm or farm. First, we selected the national forestry farm or nature reserve in that period. Then we extract the location by the ecological requirements (dense vegetation and low human influence) [1-6] using the historical remote sensing map (Google Earth).

f. Cross check

We combined all the records from gazetteers with information from the other sources. Conflicting records with unsubstantiated metadata, such as those lacking relevant or detailed descriptions, were excluded from the analysis. Again we confirmed the location with historical remote sensing map (Google Earth) according to the ecological requirements (dense vegetation and low human influence) [1-7]. Then, we divided all of the records into different periods: 1970s (1970-1979), 1980s (1980-1989), 1990s (1990-1999), 2000s (2000-2016).

5) Semi-structured interviews

Previous research indicated that such datasets can be complemented by local data after the year 2000 [11, 12, 14]. Due to the sensitivity of poaching pangolin in China, we conducted the semi-structured interviews without precise, pre-determined questions so that interesting lines of discussion could be pursued. Open questioning was employed wherever possible, to avoid leading the interviewee into an answer. All of the questions addressed the distribution, historical range, and last records for pangolins. All dialogue was recorded by one of the interviewers and uncertainties were clarified immediately after the interview.

Standards and content of semi structured interviews.

For interviewees:

a. Stay or live in mountain area for more than 10 years.

b. Often work in mountain area.

c. People once involved in pangolin trade, including poacher or doctor.

d. Able to identify pangolins from photos of multiple animal species.

e. Can distinguish between Chinese pangolin and Sudan pangolin.

Brief template of interview questions:

a. Describe the living environment and activity characteristics of pangolin.

b. When and where did you see the pangolin, especially the last time? The number of them and how often you see them. As accurate as possible.

c. The population change trend of pangolin through time.

d. Information about pangolin trade, protection policy, and other conservation action.

In total, we interviewed or commissioned interviews with 133 people. The number and time of interviews are summarized in Table S1.

Table S1 Number of interviewees and time of interviews for each city/prefecture.

| City/Prefecture | Number | Time | City/Prefecture | Number | Time |
| --- | --- | --- | --- | --- | --- |
| Pingxiang Shi | 5 | 2012/2013/2017 | Longnan Xian | 8 | 2010 |
| Guangze Xian | 2 | 2013/2017 | Nanjing Xian | 1 | 2016 |
| Shaowu Shi | 2 | 2010/2013/2017 | Pinghe Xian | 2 | 2012/2015 |
| Lichuan Xian | 2 | 2010/2013/2017 | Zhao'an Xian | 2 | 2012/2015 |
| Taining Xian | 2 | 20102013/2017 | Jian'ou Xian | 1 | 2013 |
| Jianning Xian | 2 | 2010/2013/2017 | Pingnan Xian | 1 | 2014 |
| Jiangle Xian | 2 | 2010/2013/2017 | Gutian Xian | 2 | 2013/2014 |
| Ninghua Xian | 2 | 2010/2013/2017 | Lianhua Xian | 3 | 2014 |
| Mingxi Xian | 3 | 2010/2013/2014/2017 | Longquan Shi | 2 | 2016 |
| Qingliu Xian | 3 | 2010/2013/2014/2017 | Minhou Xian | 1 | 2011 |
| Ruijin Shi | 1 | 2014 | Pucheng Xian | 2 | 2016 |
| Changting Xian | 3 | 2013/2014/2017 | Shangrao Xian | 1 | 2015 |
| Liancheng Xian | 9 | 2010 | Shangsu Xian | 3 | 2012 |
| Huichang Xian | 2 | 2013/2017 | Thousand Island Lake | 2 | 2011 |
| Chongyi Xian | 1 | 2017 | Wuzhou City | 1 | 2016 |
| Anyuan Xian | 2 | 2017 | Xiangdong Qu | 1 | 2011 |
| Longyan Shi | 2 | 2012/2013/2017 | Yongding Qu | 1 | 2011 |
| Xinfeng Xian | 8 | 2010 | Youxi Xian | 1 | 2010 |
| Wuping Xian | 1 | 2013/2017 | Yunxiao Xian | 2 | 2012/2015 |
| Shanghang Xian | 1 | 2013/2017 | Zhangping Xian | 1 | 2012 |
| Xunwu Xian | 8 | 2010 | Zhangpu Xian | 3 | 2012/2014/2015 |
| Quannan Xian | 9 | 2010/2016 | Zhoushan Shi | 9 | 2014 |
| Yongding Xian | 1 | 2012 | Xianjian Qu | 2 | 2017 |
| Dingnan Xian | 8 | 2010 | Total | 133 |  |

6) Cross check

Conflicting records with unsubstantiated metadata, such as those lacking relevant or detailed descriptions, were excluded from the analysis.

**B. Data collection**

Table S2 Records from local historical documents.

| ID | Last records | References | ID | Last records | References |
| --- | --- | --- | --- | --- | --- |
| 1 | -1990s | An'fuxian Guyuanshan Linchangzhi | 94 | -1980s | Pan'an Xianzhi |
| 2 | -2000s | An'fu Xianzhi | 95 | -1970s | Pengze Xianzhi |
| 3 | -1980s | An'ji Linyezhi | 96 | -1980s | Pingnan Xianzhi |
| 4 | -1970s | An'sha Zhengzhi | 97 | -1990s | Qingtian Xianzhi |
| 5 | -1990s | An'yi Xianzhi | 98 | -1980s | Qingyuan Quzhi |
| 6 | -1970s | An'yuan Quzhi | 99 | -1990s | Qingyuan Xianzhi |
| 7 | -1988s | An'yuanxian Linyezhi | 100 | -1980s | Quannan Xianzhi |
| 8 | -1990s | Aoqiao Xiangzhi | 101 | -1980s | Ruichang Shizhi |
| 9 | -1970s | Boyang Xianzhi | 102 | -1980s | Ruijin Xianzhi |
| 10 | -1970s | Cangnan Xianzhi | 103 | -1990s | Sanming Linyezhi |
| 11 | -1980s | Changhua Zhengzhi | 104 | -1980s | Sanqing Mountain Zhi |
| 12 | -1980s | Changjiang Quzhi | 105 | -1970s | Xiamenshi Linyezhi |
| 13 | -1985s | Changshanxian Linyezhi | 106 | -1970s | Shanggao Xianzhi |
| 14 | -1980s | Changshan Xianzhi | 107 | -1980s | Shangraoshi Nongyezhi |
| 15 | -1990s | Chengtang Zhengzhi | 108 | -1980s | Shangrao Xianzhi |
| 16 | -1980s | Chongren Xianzhi | 109 | -2000s | Shangyou Xianzhi |
| 17 | -1990s | Chongyixian Linyezhi | 110 | -1990s | Shicang Cunzhi |
| 18 | -1990s | Cuiwei Mountain Zhi | 111 | -1980s | Shicheng Xianzhi |
| 19 | -1980s | Damaoshan Zhi | 112 | -1980s | Shishi Xiangzhi |
| 20 | -1970s | Daxi Zhengzhi | 113 | -1980s | Shouningxian Linyezhi |
| 21 | -1980s | Dayu Xianzhi | 114 | -1980s | Shuangfeng Xiangzhi |
| 22 | -1970s | Daixi Zhengzhi | 115 | -1990s | Songxi Xianzhi |
| 23 | -1980s | Dexing Xianzhi | 116 | -1980s | Songyang Xianzhi |
| 24 | -1970s | Dianqian Xiangzhi | 117 | -1980s | Suichangxian Linyezhi |
| 25 | -1990s | Dingnan Xianzhi | 118 | -2000s | Suichuan Xianzhi |
| 26 | -1970s | Dongqi Cunzhi | 119 | -1970s | Taiba Xiangzhi |
| 27 | -1970s | Dongxiang Xianzhi | 120 | -1980s | Taihe Linyezhi |
| 28 | -1990s | Dongxiao Zhengzhi | 121 | -1990s | Taishun Xianzhi |
| 29 | -1970s | Dongyang Shizhi | 122 | -1970s | Tiantai Xianzhi |
| 30 | -1970s | Dongyuan Cunzhi | 123 | -1970s | Tonglu Zhengzhi |
| 31 | -1974s | Fengchengxian Linyezhi | 124 | -1990s | Tonggu Xianzhi |
| 32 | -1970s | Fengxin Xianzhi | 125 | -1990s | Wan'an Xianzhi |
| 33 | -1990s | Fuliang Xianzhi | 126 | -1980s | Wannian Xianzhi |
| 34 | -1980s | Fu'an Xianzhi | 127 | -2000s | Wanzai Xianzhi |
| 35 | -2000s | Fuyangxian Linyezhi | 128 | -1990s | Wencheng Xianzhi |
| 36 | -1980s | Ganxian Zhi | 129 | -1970s | Wutang Zhengzhi |
| 37 | -1970s | Gao'an Xianzhi | 130 | -2000s | Wuningxian Linyezhi |
| 38 | -1980s | Gutianxian Linyezhi | 131 | -1980s | Wuyixian Linyezhi |
| 39 | -1990s | Guanzhi Mountain Zhi | 132 | -1970s | Wucheng Xianzhi |
| 40 | -1990s | Guangchang Xianzhi | 133 | -1990s | Wuyuan Xianzhi |
| 41 | -1990s | Guangfeng Xianzhi | 134 | -1970s | Xianju Xianzhi |
| 42 | -1980s | Guiyang Xiangzhi | 135 | -1970s | Xianyou Linyezhi |
| 43 | -1980s | Hengfeng Xianzhi | 136 | -1980s | Xinfeng Xianzhi |
| 44 | -1990s | Hubian Zhengzhi | 137 | -1980s | Xiushui Xianzhi |
| 45 | -1980s | Hukou Xianzhi | 138 | -1970s | Xuanhe Xiangzhi |
| 46 | -1970s | Hutang Xiangzhi | 139 | -1990s | Xuwu Xianzhi |
| 47 | -1980s | Huaqiao Xiangzhi | 140 | -1990s | Yichun Shizhi |
| 48 | -1990s | Ji'an Xianzhi | 141 | -1990s | Yihuang Xianzhi |
| 49 | -1980s | Jishui Xianzhi | 142 | -1990s | Yiyang Xianzhi |
| 50 | -1970s | Jianshanxia Cunzhi | 143 | -1970s | Yiwushi Nongyezhi |
| 51 | -1975s | Jiandeshi Nongye Quhuazhi | 144 | -1990s | Yongan Shizhi |
| 52 | -1970s | Jian'ou Linyezhi | 145 | -1980s | Yongfeng Linyezhi |
| 53 | -1970s | Jianyangshi Linyezhi | 146 | -1980s | Yongfeng Xianzhi |
| 54 | -1980s | Jiangshan Shizhi | 147 | -1990s | Yongjia Xianzhi |
| 55 | -1980s | Jinxi Xianzhi | 148 | -1970s | Yongkang Xianzhi |
| 56 | -1980s | Jinyun Xianzhi | 149 | -1980s | Yongtai Xianzhi |
| 57 | -1990s | Jinggang Mountain Kenzhichang | 150 | -1990s | Yongxin Xianzhi |
| 58 | -1990s | Jinggang Mountain Zhi | 151 | -1980s | Yongxiu Xianzhi |
| 59 | -1990s | Jin'an Xianzhi | 152 | -1970s | Yudu Xianzhi |
| 60 | -1980s | Jiujiang Xianzhi | 153 | -1970s | Yuhangxian Linyezhi |
| 61 | -1970s | Junfushan Linchangzhi | 154 | -1980s | Yushan Xianzhi |
| 62 | -2000s | Kaihua Xianzhi | 155 | -1970s | Yuanzhong Cunzhi |
| 63 | -1990s | Kecheng Quzhi | 156 | -1980s | Yunhe Xianzhi |
| 64 | -1970s | Lanxishi Lvyouzhi | 157 | -1980s | Zetan Xiangzhi |
| 65 | -1970s | Le'an Xianzhi | 158 | -1990s | Zhangzhou Shizhi |
| 66 | -1980s | Leping Xianzhi | 159 | -1990s | Changting Xianzhi |
| 67 | -1980s | Lishan Linchangzhi | 160 | -1970s | Zherong Xianzhi |
| 68 | -1980s | Liancheng Xianzhi | 161 | -2000s | Zhejiang Province Linyezhi |
| 69 | -1990s | Lianhua Xianzhi | 162 | -1990s | Zhenghe Xianzhi |
| 70 | -1970s | Lin'an Linyezhi | 163 | -1975s | Zhouning Xianzhi |
| 71 | -1970s | Linchuan Quzhi | 164 | -1970s | Zhukeng Xianzhi |
| 72 | -1980s | Lingshan Mountain Zhi | 165 | -2000s | Zixi Linyezhi |
| 73 | -1980s | Longhu Mountain Zhi | 166 | -2000s | Ninghua Xianzhi |
| 74 | -2000s | Longnan Xianzhi | 167 | -2000s | Guangze Xianzhi |
| 75 | -1990s | Longquanshi Linyezhi | 168 | -2000s | Fangxi Xianzhi |
| 76 | -1990s | Long Mountain Zhi | 169 | -2000s | Lichuan Xianzhi |
| 77 | -1980s | Longyou Xianzhi | 170 | -2000s | Congyixian Linyezhi |
| 78 | -1980s | Luxiling Linchangzhi | 171 | -2000s | Huichang Xianzhi |
| 79 | -1985s | Luxi Xianzhi | 172 | -2000s | Jing'an Xianzhi |
| 80 | -1970s | Lucheng Quzhi | 173 | -2000s | Guanshan Xianzhi |
| 81 | -1970s | Luoyuan Xianzhi | 174 | -1980s | Putian Xianzhi |
| 82 | -1980s | Luokou Zhengzhi | 175 | -1970s | Jiaocheng Xianzhi |
| 83 | -1990s | Matou Mountain Linchangzhi | 176 | -1970s | Xinzhou Quzhi |
| 84 | -1980s | Minqing Xianzhi | 177 | -1980s | Leqing Shizhi |
| 85 | -1970s | Mogan Mountain Zhi | 178 | -1990s | Qujiang Quzhi |
| 86 | -1980s | Nan'an Xianzhi | 179 | -1990s | Liandu Quzhi |
| 87 | -1970s | Nanchangshi Nongyezhi | 180 | -1980s | Longquan Shizhi |
| 88 | -1980s | Nancheng Xianzhi | 181 | -1980s | Duchang Xianzhi |
| 89 | -1980s | Nanfeng Xianzhi | 182 | -1980s | Yugan Xianzhi |
| 90 | -1980s | Nankang Xianzhi | 183 | -1990s | Shunchang Xianzhi |
| 91 | -1980s | Nanping Xianzhi | 184 | -1990s | Mingxi Xianzhi |
| 92 | -1980s | Ningdu Linyezhi | 185 | -1990s | Shaowu Shizhi |
| 93 | -1970s | Panshan Cunzhi | 186 | -1990s | Wuyi Shizhi |

Table S3 Records from other resources

| ID | Last record | Location | Reference |
| --- | --- | --- | --- |
| 1 | 2000s | Chongyi Xian | Interview Information from Local People |
| 2 | 2000s | Chongyi Xian | Interview Information from Local People |
| 3 | 2000s | Chongyi Xian | Interview Information from Local People |
| 4 | 2000s | Chongyi Xian | Interview Information from Local People |
| 5 | 2000s | Chongyi Xian | Interview Information from Local People |
| 6 | 2000s | Chun'an Xian | News |
| 7 | 1980s | Duchang Xian | Article[17] |
| 8 | 1980s | Duchang Xian | Article[17] |
| 9 | 1980s | Fuyang Qu | Article[18] |
| 10 | 1970s | Fuyang Qu | Article[18] |
| 11 | 2000s | Jianning Xian | Scientific Investigation for Nature Reserve |
| 12 | 2000s | Jianning Xian | Personal Communication |
| 13 | 2000s | Jinggang Mountain Shi | Scientific Investigation for Nature Reserve |
| 14 | 2000s | Jinggang Mountain Shi | Scientific Investigation for Nature Reserve |
| 15 | 2000s | Jinggang Mountain Shi | Scientific Investigation for Nature Reserve |
| 16 | 2000s | Jinggang Mountain Shi | Scientific Investigation for Nature Reserve |
| 17 | 2000s | Liancheng Xian | Interview Information from Local People |
| 18 | 2000s | Liancheng Xian | Interview Information from Local People |
| 19 | 2000s | Liancheng Xian | Interview Information from Local People |
| 20 | 2000s | Lianhua Xian | Interview Information from Local People |
| 21 | 2000s | Longquan Shi | Interview Information from Local People |
| 22 | 2000s | Longquan Shi | Interview Information from Local People |
| 23 | 1980s | Lushan Mountain | Article[17] |
| 24 | 1970s | Minhou Xian | Interview Information from Local People |
| 25 | 1990s | Ninghua Xian | Interview Information from Local People/Article[19] |
| 26 | 2000s | Pucheng Xian | Interview Information from Local People/Article[19] |
| 27 | 2000s | Pucheng Xian | Interview Information from Local People/Article[19] |
| 28 | 2000s | Pucheng Xian | Interview Information from Local People/Article[19] |
| 29 | 2000s | Pucheng Xian | Interview Information from Local People/Article[19] |
| 30 | 1990s | Thousand Island Lake | Interview Information from Local People/Article[20] |
| 31 | 2000s | Ruijin Xian | Interview Information from Local People |
| 32 | 2000s | Ruijin Xian | Interview Information from Local People/Article [8] |
| 33 | 2000s | Ruijin Xian | Interview Information from Local People/Article [8] |
| 34 | 2000s | Ruijin Xian | Interview Information from Local People/Article [8] |
| 35 | 2000s | Shanghang Xian | Interview Information from Local People/Article[19] |
| 36 | 2000s | Shanghang Xian | Interview Information from Local People/Article[19] |
| 37 | 2000s | Shanghang Xian | Interview Information from Local People/Article[19] |
| 38 | 2000s | Shanghang Xian | Interview Information from Local People/Article[19] |
| 39 | 2000s | Shanghang Xian | Interview Information from Local People/Article[19] |
| 40 | 2000s | Shanghang Xian | Interview Information from Local People/Article[19] |
| 41 | 2000s | Shangrao Xian | Interview Information from Local People |
| 42 | 1980s | Shangsu Xian | Interview Information from Local People |
| 43 | 1980s | Shangsu Xian | Interview Information from Local People |
| 44 | 2000s | Taining Xian | Personal Communication |
| 45 | 2000s | Taining Xian | Personal Communication |
| 46 | 2000s | Taining Xian | Personal Communication |
| 47 | 2000s | Wuyanling | Scientific Investigation for Nature Reserve |
| 48 | 2000s | Wuyanling | Scientific Investigation for Nature Reserve |
| 49 | 2000s | Wuyanling | Scientific Investigation for Nature Reserve |
| 50 | 2000s | Fuzhou City | Interview Information from Local People |
| 51 | 2000s | Wuping Xian | Interview Information from Local People |
| 52 | 2000s | Wuping Xian | Interview Information from Local People |
| 53 | 2000s | Wuping Xian | Interview Information from Local People |
| 54 | 2000s | Wuping Xian | Interview Information from Local People |
| 55 | 2000s | Wuping Xian | Interview Information from Local People |
| 56 | 2000s | Wuping Xian | Interview Information from Local People |
| 57 | 2000s | Wuping Xian | Interview Information from Local People |
| 58 | 2000s | Wuyi Mountain | Scientific Investigation for Nature Reserve/Article[19] |
| 59 | 2000s | Wuyi Mountain | Scientific Investigation for Nature Reserve/Article[19] |
| 60 | 2000s | Wuyi Mountain | Scientific Investigation for Nature Reserve/Article[19] |
| 61 | 2000s | Wuyi Mountain | Scientific Investigation for Nature Reserve/Article[19] |
| 62 | 2000s | Wuyuan Xian | Scientific Investigation for Nature Reserve |
| 63 | 2000s | Wuyuan Xian | Scientific Investigation for Nature Reserve |
| 64 | 2000s | Wuyuan Xian | Scientific Investigation for Nature Reserve |
| 65 | 2000s | Xiangdong Qu | Interview Information from Local People |
| 66 | 2000s | Xiangdong Qu | Interview Information from Local People |
| 67 | 2000s | Xiangdong Qu | Interview Information from Local People |
| 68 | 2000s | Xiangdong Qu | Interview Information from Local People |
| 69 | 2000s | Xinjian Qu | Interview Information from Local People |
| 70 | 1980s | Xingzi Xian | Article[17] |
| 71 | 2000s | Yongding Qu | Interview Information from Local People/Article[19] |
| 72 | 2000s | Yongding Qu | Interview Information from Local People/Article[19] |
| 73 | 1980s | Yongxiu Xian | Article[17] |
| 74 | 1980s | Yongxiu Xian | Article[17] |
| 75 | 1980s | Yongxiu Xian | Article[17] |
| 76 | 1970s | Youxi Xian | Interview Information from Local People/Article[19] |
| 77 | 1970s | Youxi Xian | Interview Information from Local People/Article[19] |
| 78 | 1970s | Youxi Xian | Interview Information from Local People/Article[19] |
| 79 | 1990s | Yunxiao Xian | Interview Information from Local People |
| 80 | 1980s | Zhangping Xian | Interview Information from Local People/Article[19] |
| 81 | 1990s | Zhangping Xian | Interview Information from Local People/Article[19] |
| 82 | 1990s | Zhangpu Xian | Interview Information from Local People/Article[19] |
| 83 | 2000s | Changting Xian | Interview Information from Local People/Article[19] |
| 84 | 2000s | Changting Xian | Interview Information from Local People/Article[19] |
| 85 | 2000s | Changting Xian | Interview Information from Local People/Article[19] |
| 86 | 2000s | Changting Xian | Interview Information from Local People/Article[19] |
| 87 | 2000s | Changting Xian | Interview Information from Local People/Article[19] |
| 88 | 2000s | Changting Xian | Interview Information from Local People/Article[19] |
| 89 | 2000s | Changting Xian | Interview Information from Local People/Article[19] |
| 90 | 1990s | Changting Xian | Interview Information from Local People/Article[19] |
| 91 | 1990s | Zhao'an Xian | Interview Information from Local People |
| 92 | 1990s | Zhao'an Xian | Interview Information from Local People |
| 93 | 2000s | Zhoushan Shi | Interview Information from Local People/Article[21, 22] |
| 94 | 2000s | Zhoushan Shi | Interview Information from Local People/Article[21, 22] |

**C. Home Range of Chinese Pangolin**

Home range is the area, usually around a home site, over which the animal normally travels in search of food[23]. Body size, metabolic needs, social organization, and behavior can affect the animal's home range size[24, 25]. Unfortunately, there are few information on the home range of Chinese pangolin.

For two reasons:

1. the relationship between *Manis javanica* and *Manis pentadactyla* is most closed in Manidae.

2. the heredity, morphology, and the behaviors of these two species are similar. Last but not least, there are overlapping areas between these two species’ habitat in Southern China[26-29].

Therefore, we use home range of the Sunda pangolin, *Manis javanica* for analysis, which is 6.97 ha[30].

**Reference**

[1] WU, S., MA, G., TANG, M., CHEN, H. & LIU, N. 2002 The status and conservation strategy of pangolin resource in China. *Journal of Natural Resources* **17**, 174-180. (doi:10.3321/j.issn:1000-3037.2002.02.008).

[2] WU, S., MA, G., TANG, M., CHEN, H., XU, Z. & LIU, N. 2002 The population and density of pangolin in dawuling natural reserve and the number of pangolin resource in guangdong province. *Acta Theriologica Sinica* **22**, 270-276. (doi:10.3969/j.issn.1000-1050.2002.04.005).

[3] Wu, S.B., LIU, N., MA, G.Z., TANG, M., CHEN, H. & XU, Z. 2004 A Current Situation of Ecology Study on Pangolins. *Chinese Journal of Zoology* **39**, 46-52. (doi:10.3969/j.issn.0250-3263.2004.02.010).

[4] Wu, S.B., LIU, N., Li, Y. & Sun, R. 2005 Observation on food habits and foraging behavior of Chinese Pangolin (*Manis Pentadactyla*). *Chinese Journal of Applied & Environmental Biology* **11**, 337-341. (doi:10.3321/j.issn:1006-687X.2005.03.019).

[5] Newton, P., Nguyen, T.V., Roberton, S. & Bell, D. 2008 Pangolins in peril: using local hunters knowledge to conserve elusive species in Vietnam. *Endangered Species Research* **6**, 41-53. (doi:10.3354/esr00127).

[6] Bao, F.Y., Wu, S.B., Su, C., Yang, L., Zhang, F.H. & Ma, G.Z. 2013 Air temperature changes in a burrow of Chinese pangolin, Manis pentadactyla, in winter. *Folia Zool* **62**, 42-47.

[7] Fan, C. 2005 Burrow Habitat of Formosan Pangolins (*Manis pentadactyla pentadactyla*) at Feitsui Reservoir. Taipei, Taiwan University.

[8] Jiang, Z., Ma, Y., Wu, Y., Wang, Y., Zhou, K., Liu, S. & Feng, Z. 2015 *China's Mammal Diversity and Geographic Distribution*. Beijing, Science Press.

[9] Turvey, S.T., Crees, J.J. & Di Fonzo, M.M.I. 2015 Historical data as a baseline for conservation: reconstructing long-term faunal extinction dynamics in Late Imperial–modern China. *Proceedings of the Royal Society B: Biological Sciences* **282**, 20151299. (doi:10.1098/rspb.2015.1299).

[10] Ren, Y., Yang, L., Zhang, R., Lv, J., Huang, M. & Luan, X. 2016 Decline and Range Contraction of Black-Billed Capercaillie (*Tetrao urogalloides*) in Northeast China from 1950 to 2010 Based on Local Historical Documents. *Pakistan Journal of Zoology* **48**, 1825-1830.

[11] Yang, L., Huang, M., Zhang, R., Jiang, L., Ren, Y., Jiang, Z., Zhang, W. & Luan, X.F. 2016 Reconstructing the historical distribution of the Amur Leopard (*Panthera pardus orientalis*) in Northeast China based on historical records. *ZooKeys* **592**, 143-153. (doi:10.3897/zookeys.592.6912).

[12] Yang, L., Zhang, R., Duo, H., Zhang, W., Jiang, Z., Ren, Y., Lv, J., Huang, M., Liu, F., Shahid, M., et al. 2017 Historical distribution of lynx (*Lynx lynx*) in Northeast China on the basis of historical records. *Russian Journal of Ecology* **48**, 569-582. (doi:10.1134/S1067413617060133).

[13] Zhang, R., Yang, L., Ai, L., Yang, Q., Chen, M., Li, J., Yang, L. & Luan, X. 2017 Geographic characteristics of sable (*Martes zibellina*) distribution over time in Northeast China. *Ecology and Evolution* **00**, 1-8. (doi:10.1002/ece3.2983).

[14] Zhang, R., Yang, L., Laguardia, A., Jiang, Z., Huang, M., Lv, J., Ren, Y., Zhang, W. & Luan, X. 2016 Historical distribution of the otter (*Lutra lutra*) in north-east China according to historical records (1950-2014). *Aquatic Conservation: Marine and Freshwater Ecosystems* **26**, 602-606. (doi:10.1002/aqc.2624).

[15] Challender, D., Baillie, J., Ades, G., Kaspal, P., Chan, B., Khatiwada, A., Xu, L., Chin, S., KC, R., Nash, H., et al. 2014 *Manis pentadactyla*. In *The IUCN Red List of Threatened Species* (ed. IUCN), p. e.T12764A45222544. UK.

[16] Wen, R. 2009 *The distributions and changes of rare wild animals in China*. Chongqing, Chongqing Science and Technology Press.

[17] Fu, D. & Ding, T. 1991 Mammals survey in Poyang Area. *Chinese Journal of Zoology* **26**, 27-31. (doi:10.13859/j.cjz.1991.02.010).

[18] Zhu, X. & Yang, C. 1984 Preliminary investigation for terrestrial vertebrate in northwest an'ji xian. *Journal of Zhejiang Forestry College* **1**, 119-120.

[19] Zhou, D. 1996 Present situation and countermeasures of the protection and management of *Manis Pentadactyla* in Fujian Province. *Journal of Fujian Forestry Science & Technology* **23**, 85-88. (doi:10.13428/j.cnki.fjlk.1996.02.019).

[20] Xu, A., Si, X., Wang, Y. & Ding, P. 2014 Camera traps and the minimum trapping effort for ground-dwelling mammals in fragmented habitats in the Thousand Island Lake, Zhejiang Province. *Biodiversity Science* **22**, 764-772. (doi:10.3724/SP.J.1003.2014.14176).

[21] Li, Y. & Li, D. 1994 Changes of natural habitats on Zhoushan Island and their effects on species extinction of mammals. *Chinese Journal of Applied Ecology* **5**, 269-275. (doi:10.13287/j.1001-9332.1994.0051).

[22] Zhu, X., Cao, W. & Wang, J. 2010 Mammalian fauna and distribution of Putuoshan Island in Zhoushan. *Journal of Zhejiang Forestry College* **27**, 110-115. (doi:10.3969/j.issn.2095-0756.2010.01.018).

[23] Burt, W.H. 1943 Territoriality and Home Range Concepts as Applied to Mammals. *Journal of Mammalogy* **24**, 346-352.

[24] Gittleman, J.L. & Harvey, P.H. 1982 Carnivore Home-Range Size, Metabolic Needs and Ecology. *Behavioral Ecology & Sociobiology* **10**, 57-63.

[25] Lindstedt, S.L., Miller, B.J. & Buskirk, S.W. 1986 Home Range, Time, and Body Size in Mammals. *Ecology* **67**, 413-418.

[26] Gaudin, T.J., Emry, R.J. & Wible, J.R. 2009 The Phylogeny of Living and Extinct Pangolins (Mammalia, Pholidota) and Associated Taxa: A Morphology Based Analysis. *Journal of Mammalian Evolution* **16**, 235.

[27] Hassanin, A., Hugot, J.P. & van Vuuren, B.J. 2015 Comparison of mitochondrial genome sequences of pangolins (Mammalia, Pholidota). *Comptes Rendus Biologies* **338**, 260-265.

[28] Jiang, H., Feng, M. & Huang, J. 1988 Pangolin--Preliminary observation of activity habits. Chinese Journal of Wildlife, 11-13.

[29] Wu, S., Wang, Y. & Feng, Q. 2005 A New Record of Chinese Mammals--Manis javanica. Zoological Systematics 30, 440-443. (doi:10.3969/j.issn.1000-0739.2005.02.039).

[30] Lim, N.T.L. & Ng, P.K.L. 2008 Home range, activity cycle and natal den usage of a female Sunda pangolin Manis javanica (Mammalia: Pholidota) in Singapore. Endangered Species Research 4, 233-240.
