# Supplementary material for: Historical data for conservation: reconstructing range changes of Chinese pangolin (Manis pentadactyla) in eastern China (1970–2016)
Source: Proc Biol Sci. 2018 Aug 22;285(1885):20181084. doi: 10.1098/rspb.2018.1084 (PMC6125891; doi:10.1098/rspb.2018.1084)
Supplement: Appendix II Environmental variables, niche analysis and model performance [file rspb20181084supp2.docx]

*Historical data for conservation: reconstructing range changes of Chinese pangolin (Manis pentadactyla) in eastern China (1970-2016)*

Li Yang, Minhao Chen, Daniel W.S. Challender, Carly Waterman, Chao Zhang, Zhaomin Huo, Hongwei Liu, Xiaofeng Luan

**Appendix II** **Environmental variables, niche analysis and model performance**

**A. Environmental variable selection**

Historical climate data can be obtained by interpolating complex multivariate data using thin plate smoothing splines. First, climate data from 1970−2015 were downloaded from Climate AP v2.03 (http://climateap.net/;[1]). According to previous research [2-7], temperature bioclimatic variables (MAT, mean annual temperature, °C), precipitation bioclimatic variables (MAP, mean annual precipitation, mm), two derived variables (NFFD, the number of frost-free days; Eref, Hargreaves reference evaporation) and topography (elevation and aspect) can be vital to pangolins and their prey. Four bioclimatic variables (MAT, MAP, NFFD, Eref) were calculated over 10-year periods using ANUSPLIN ver. 4.36, and topography data (elevation and aspect) were obtained from the SRTM 90m Digital Elevation Database (http://srtm.csi.cgiar.org/).

Pangolins are affected by human disturbance [3, 8]. However, this influence is hard to quantify and include in analyses because historical data on human activities from the 1970s are often unavailable and difficult to compile. Therefore, two variables (forested and urban land) were extracted from land cover data since the 1970s (dataset provided by the Data Center for Resources and Environmental Sciences, Chinese Academy of Sciences [RESDC; http://www.resdc.cn]). All variables were obtained at the same spatial resolution of 30 s. A total of eight variables were obtained for further research.

**B. Niche analysis**

First, the dataset representing the total environmental space was created using all variables as measured for 10,000 background points drawn for each time frame. The first two axes of a PCA calibrated over the entire range of possible environments in the study area were created by the total environmental space. For the total niche space, we pooled all occurrence data through time, exacted the values from the variables corresponding to locations and time frames, and projected them into the PCA space. We followed the same process for each time frame (from the 1970s to the 2000s) separately, and then projected all of the points of presence for each period into the total PCA space to delineate the partial niche space for each period. Then, we compared the partial niches with the total niche following Broennimann et al. [9] and tested whether the similarity of the partial niches to the total niche was greater than chance using Schoener’s D (an index of overlap, Niche Similarity Test in R package “ecospat”). Different numbers of occurrences between total and partial niches can cause bias. Therefore, we randomly resampled the same amount of occurrence data for the total niche as we had available for a given partial niche. We repeated the analysis for 100 subsamples of the total niche, thus obtaining a dataset of 100 Schoener’s D values and *P*-values for the niche similarity test for each period.

**B. Model performance**

Table S1. Eco-geographic variables used in species distribution models of Chinese pangolin in eastern China for different periods

| ID | Describe | Name | Resource | 1970s | 1980s | 1990s | 2000s |
| --- | --- | --- | --- | --- | --- | --- | --- |
| 1 | Mean annual temperature | MAT | Climate AP | 8.07 | EX | 7.35 | 6.98 |
| 2 | Mean annual precipitation | MAP | Climate AP | 2.33 | 2.19 | 1.68 | 1.92 |
| 3 | The number of frost-free days | NFFD | Climate AP | 4.44 | 1.75 | 3.98 | 3.88 |
| 4 | Hargreaves reference evaporation | Eref | Climate AP | 3.05 | 1.91 | 2.52 | 2.17 |
| 5 | Elevation | Elevation | SRTM 90m Digital Elevation Database | 2.63 | 2.15 | 2.44 | 2.52 |
| 6 | Aspect | Aspect | SRTM 90m Digital Elevation Database | 1.00 | 1.00 | 1.00 | 1.00 |
| 7 | Urban land | Urban land | RESDC | 1.37 | 1.36 | 1.37 | 1.37 |
| 8 | Forestry | Forestry | RESDC | 1.15 | 1.17 | 1.17 | 1.18 |

NOTE: Period 1970s is 1970-1979; 1980s is 1980-1989; 1990s is 1990-1999; 2000s is 2000-2016. EX means that the variable is excluded by the VIF.

Climate AP v2.03, http://climateap.net/

SRTM 90m Digital Elevation Database, http://srtm.csi.cgiar.org/

Data Center for Resources and Environmental Sciences, Chinese Academy of Sciences (RESDC), http://www.resdc.cn

Table S2. The true skill statistic (TSS) calculated for each model for different periods

|  | 1970s | 1980s | 1990s | 2000s |
| --- | --- | --- | --- | --- |
| GAM | 0.40±0.03 | 0.45±0.02 | 0.50±0.04 | 0.57±0.05 |
| GLM | 0.42±0.03 | 0.46±0.02 | 0.53±0.05 | 0.56±0.04 |
| ANN | 0.35±0.09 | 0.41±0.06 | 0.47±0.03 | 0.49±0.08 |
| GBM | 0.42±0.03 | 0.48±0.01 | 0.55±0.04 | 0.60±0.05 |
| FDA | 0.41±0.03 | 0.44±0.03 | 0.50±0.06 | 0.52±0.03 |
| MARS | 0.42±0.03 | 0.45±0.03 | 0.51±0.05 | 0.56±0.07 |
| RF | 0.42±0.02 | 0.48±0.03 | 0.53±0.04 | 0.57±0.06 |
| MAXENT.Phillips | 0.41±0.03 | 0.45±0.03 | 0.51±0.05 | 0.55±0.05 |

Table S3. Relative importance of variables for Chinese pangolin in eastern China for different periods

| Variables | 1970s | 1980s | 1990s | 2000s |
| --- | --- | --- | --- | --- |
| Aspect | 0.017 | 0.010 | 0.013 | 0.013 |
| Elevation | 0.642 | 0.349 | 0.508 | 0.727 |
| Eref | 0.055 | 0.100 | 0.022 | 0.083 |
| Forestry | 0.066 | 0.074 | 0.141 | 0.028 |
| Map | 0.014 | 0.028 | 0.051 | 0.030 |
| Mat | 0.074 | - | 0.064 | 0.135 |
| NFFD | 0.317 | 0.160 | 0.084 | 0.356 |
| Urban land | 0.026 | 0.059 | 0.089 | 0.068 |

Note: the grey color shows the first three variables.

**Reference**

[1] Wang, T., Hamann, A., Spittlehouse, D.L. & Murdock, T.Q. 2012 ClimateWNA—High-Resolution Spatial Climate Data for Western North America. *Journal of Applied Meteorology and Climatology* **51**, 16-29. (doi:10.1175/jamc-d-11-043.1).

[2] WU, S., MA, G., TANG, M., CHEN, H. & LIU, N. 2002 The status and conservation strategy of pangolin resource in China. *Journal of Natural Resources* **17**, 174-180. (doi:10.3321/j.issn:1000-3037.2002.02.008).

[3] WU, S., MA, G., TANG, M., CHEN, H., XU, Z. & LIU, N. 2002 The population and density of pangolin in dawuling natural reserve and the number of pangolin resource in guangdong province. *Acta Theriologica Sinica* **22**, 270-276. (doi:10.3969/j.issn.1000-1050.2002.04.005).

[4] Wu, S.B., LIU, N., MA, G.Z., TANG, M., CHEN, H. & XU, Z. 2004 A Current Situation of Ecology Study on Pangolins. *Chinese Journal of Zoology* **39**, 46-52. (doi:10.3969/j.issn.0250-3263.2004.02.010).

[5] Fan, C. 2005 Burrow Habitat of Formosan Pangolins (*Manis pentadactyla pentadactyla*) at Feitsui Reservoir. Taipei, Taiwan University.

[6] Wu, S.B., LIU, N., Li, Y. & Sun, R. 2005 Observation on food habits and foraging behavior of Chinese Pangolin (*Manis Pentadactyla*). *Chinese Journal of Applied & Environmental Biology* **11**, 337-341. (doi:10.3321/j.issn:1006-687X.2005.03.019).

[7] Bao, F.Y., Wu, S.B., Su, C., Yang, L., Zhang, F.H. & Ma, G.Z. 2013 Air temperature changes in a burrow of Chinese pangolin, Manis pentadactyla, in winter. *Folia Zool* **62**, 42-47.

[8] Xu, A., Si, X., Wang, Y. & Ding, P. 2014 Camera traps and the minimum trapping effort for ground-dwelling mammals in fragmented habitats in the Thousand Island Lake, Zhejiang Province. *Biodiversity Science* **22**, 764-772. (doi:10.3724/SP.J.1003.2014.14176).

[9] Broennimann, O., Fitzpatrick, M.C., Pearman, P.B., Petitpierre, B., Pellissier, L., Yoccoz, N.G., Thuiller, W., Fortin, M.-J., Randin, C., Zimmermann, N.E., et al. 2012 Measuring ecological niche overlap from occurrence and spatial environmental data. *Global Ecology and Biogeography* **21**, 481-497. (doi:10.1111/j.1466-8238.2011.00698.x).
