# Supplementary material for: Historical data for conservation: reconstructing range changes of Chinese pangolin (Manis pentadactyla) in eastern China (1970–2016)
Source: Proc Biol Sci. 2018 Aug 22;285(1885):20181084. doi: 10.1098/rspb.2018.1084 (PMC6125891; doi:10.1098/rspb.2018.1084)
Supplement: Appendix III Conservation Prioritization [file rspb20181084supp3.docx]

*Historical data for conservation: reconstructing range changes of Chinese pangolin (Manis pentadactyla) in eastern China (1970-2016)*

Li Yang, Minhao Chen, Daniel W.S. Challender, Carly Waterman, Chao Zhang, Zhaomin Huo, Hongwei Liu, Xiaofeng Luan

**Appendix III Conservation Prioritization**

**A. Cost layer**

According to Zonation 4.00rc1, the cost layer is associated with the cost of protecting the particular site.

Here, we assumed that the time of animal disappearance was associated with the cost: the longer it is absent, the harder it is to recover the population.

How to create cost layer?

1) obtain the SDMs results for each period. The layer contains values ranged from 0 to 1 (probability value, “0” means absent, “1” means present). This is model result from BIOMOD2. Then convert the value into 1 to 0 (probability value, “1” means absent, “0” means present).

2) overlay the SDMs results.

3) obtain cost layer by average.

$$Cost layer=\frac{\left( {Layer}_{1970s}+{Layer}_{1980s}+{Layer}_{1990s}+{Layer}_{2000s} \right)}{4}$$

Therefore, values of cost layer ranged from 0 to 1. Value near “0” means that pangolin have high probability to maintain in this cell for more than 40 years; and value tend to be “1” means that pangolin seem to be absent since the 1970s. Because the longer pangolin is absent, the more cost we should take to recover the population.

The spatial analyses were conducted in ArcGIS (ver. 10.2; ESRI, Inc., Redlands, CA, USA).

**B. Conservation priority analysis by Zonation**


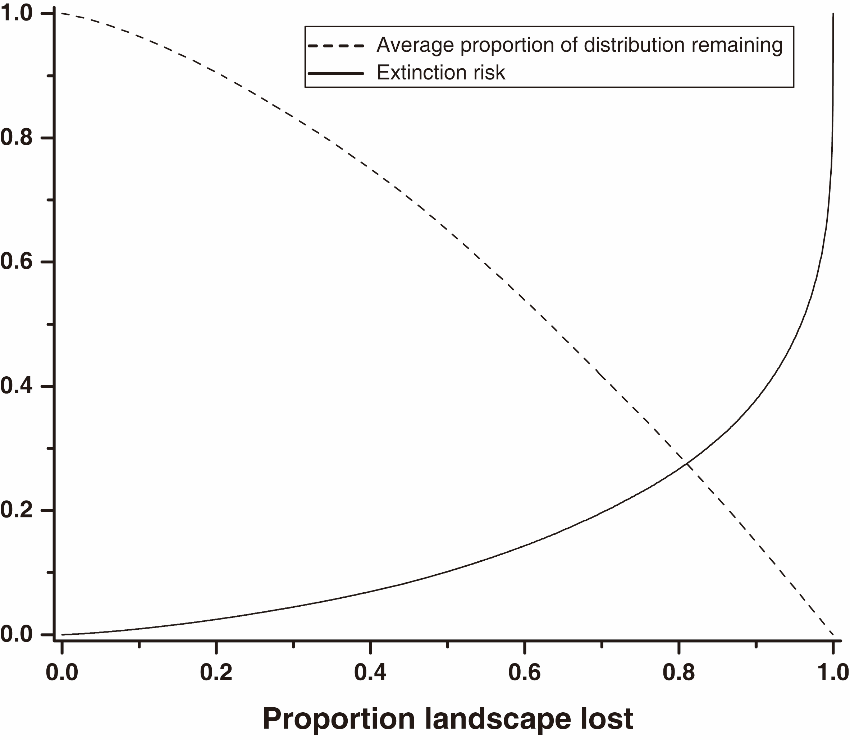


Figure S1 the average proportion of biodiversity feature distributions remaining as landscape is removed (show as dash line); and average extinction risk calculated from the canonical species-area curve (show as black line).


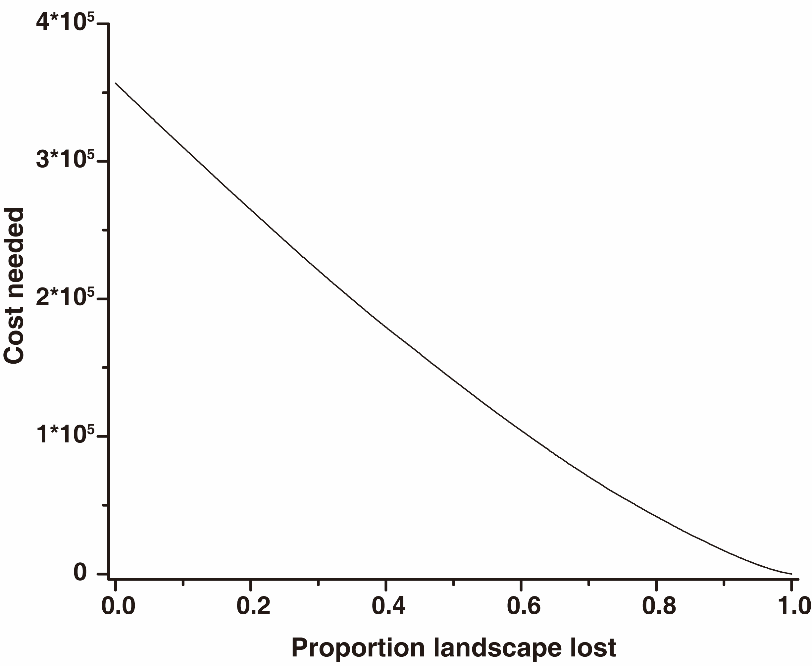


Figure S2 Cost needed to achieve a given conservation value (the number of cells needed for the respective top fractions).

**C. Nature Reserves**

Table S1 the basic information for nature reserves in study area

| ID | Name | Province | Area(km^2^) | Type | Level | Quadrant |
| --- | --- | --- | --- | --- | --- | --- |
| 1 | Anxi Yunzhongshan | Fujian | 40.95 | Forest Ecosystem | Provincial | C |
| 2 | Daiyunshan | Fujian | 134.72 | Forest Ecosystem | National | C |
| 3 | Fujian Wuyishan | Fujian | 565.27 | Forest Ecosystem | National | A |
| 4 | Huboliao | Fujian | 26.50 | Forest Ecosystem | National | EX |
| 5 | Jiangshi | Fujian | 11.87 | Forest Ecosystem | Provincial | D |
| 6 | Junzifeng | Fujian | 180.61 | Forest Ecosystem | National | A |
| 7 | Longqishan | Fujian | 156.93 | Forest Ecosystem | National | A |
| 8 | Mangdangshan | Fujian | 94.42 | Forest Ecosystem | National | C |
| 9 | Meihuashan | Fujian | 221.68 | Forest Ecosystem | National | A |
| 10 | Minjiangyuan | Fujian | 130.22 | Forest Ecosystem | National | A |
| 11 | Qingliu Lianhuashan | Fujian | 17.22 | Forest Ecosystem | Provincial | A |
| 12 | Tianbaoyan | Fujian | 110.15 | Forest Ecosystem | National | A |
| 13 | Tingjiangyuan | Fujian | 103.80 | Forest Ecosystem | National | A |
| 14 | Xiongjiang Huangchulin | Fujian | 125.13 | Forest Ecosystem | National | EX |
| 15 | Ganjiangyuan | Jiangxi | 161.01 | Forest Ecosystem | National | A |
| 16 | Guanshan | Jiangxi | 115.01 | Forest Ecosystem | National | A |
| 17 | Jiangxi Jiulingshan | Jiangxi | 115.41 | Forest Ecosystem | National | C |
| 18 | Jiangxi Matoushan | Jiangxi | 138.67 | Forest Ecosystem | National | A |
| 19 | Jiangxi Wuyishan | Jiangxi | 160.07 | Forest Ecosystem | National | A |
| 20 | Jinggangshan | Jiangxi | 214.99 | Forest Ecosystem | National | C |
| 21 | Jiulianshan | Jiangxi | 134.12 | Forest Ecosystem | National | C |
| 22 | Lushan | Jiangxi | 201.20 | Forest Ecosystem | National | C |
| 23 | Nanfengmian | Jiangxi | 42.05 | Forest Ecosystem | Provincial | A |
| 24 | Poyanghuhouniao | Jiangxi | 224.00 | Endangered Species (Animal) | National | EX |
| 25 | Poyanghunanjishidi | Jiangxi | 333.00 | Inland Wetland | National | EX |
| 26 | Qiyunshan | Jiangxi | 171.05 | Forest Ecosystem | National | C |
| 27 | Taohongling Meihualu | Jiangxi | 125.00 | Endangered Species (Animal) | National | EX |
| 28 | Tongboshan | Jiangxi | 108.00 | Forest Ecosystem | National | A |
| 29 | Yangjifeng | Jiangxi | 109.46 | Endangered Species (Animal) | National | A |
| 30 | Yihuang Huananhu | Jiangxi | 583.00 | Endangered Species (Animal) | Provincial | C |
| 31 | Anjixiaoni | Zhejiang | 12.42 | Endangered Species (Animal) | Provincial | C |
| 32 | Dapanshan | Zhejiang | 45.58 | Endangered Species (Plant) | National | EX |
| 33 | Fengyangshan-Baishanzu | Zhejiang | 260.52 | Forest Ecosystem | National | A |
| 34 | Gutianshan | Zhejiang | 81.08 | Endangered Species (Animal) | National | A |
| 35 | Lin'an Qingliangfeng | Zhejiang | 108.00 | Forest Ecosystem | National | C |
| 36 | Wuyanling | Zhejiang | 188.62 | Forest Ecosystem | National | C |
| 37 | Xianju Kuocangshan | Zhejiang | 27.01 | Forest Ecosystem | Provincial | C |
| 38 | Zhejiang Jiulongshan | Zhejiang | 55.25 | Forest Ecosystem | National | A |
| 39 | Zhejiang Tianmushan | Zhejiang | 42.84 | Endangered Species (Plant) | National | C |

Note: The information of nature reserves come from the Ministry of Environmental Protection of the People’s Republic of China (http://www.zhb.gov.cn/). Quadrant is associated to Figure 5 in the article. A means the priority nature reserve (46.15%); D means the important nature reserve (2.56%); C means the normal nature reserve (35.90%); EX means the nature reserve was excluded based on the 2000s (15.38%).

**D. Prefectures**

Table S2 the basic information for prefectures in study area

| NO. | Prefecture | Quadrant | NO. | Prefecture | Quadrant |
| --- | --- | --- | --- | --- | --- |
| 1 | AnfuXian | C | 117 | PingheXian | C |
| 2 | AnjiXian | C | 118 | PinghuShi | EX |
| 3 | AnxiXian | C | 119 | PingnanXian | A |
| 4 | AnyiXian | EX | 120 | PingtanXian | EX |
| 5 | AnyuanXian | A | 121 | PingxiangShiShixiaqu | C |
| 6 | BoyangXian | C | 122 | PingyangXian | EX |
| 7 | CangnanXian | EX | 123 | PuchengXian | A |
| 8 | ChangleShi | EX | 124 | PujiangXian | C |
| 9 | ChangshanXian | D | 125 | PutianShiShixiaqu | EX |
| 10 | ChangtaiXian | C | 126 | PutianXian | EX |
| 11 | ChangtingXian | A | 127 | QingliuXian | A |
| 12 | ChangxingXian | EX | 128 | QingtianXian | D |
| 13 | ChongrenXian | C | 129 | QingyuanXian | A |
| 14 | ChongyiXian | D | 130 | QuannanXian | C |
| 15 | ChunanXian | D | 131 | QuanzhouShiShixiaqu | EX |
| 16 | CixiShi | EX | 132 | QuXian | A |
| 17 | DaishanXian | EX | 133 | QuzhouShiShixiaqu | EX |
| 18 | DatianXian | D | 134 | RuianShi | C |
| 19 | DayuXian | C | 135 | RuichangShi | C |
| 20 | DeanXian | EX | 136 | RuijinShi | A |
| 21 | DehuaXian | D | 137 | SanmenXian | EX |
| 22 | DeqingXian | EX | 138 | SanmingShiShixiaqu | D |
| 23 | DexingShi | C | 139 | ShanggaoXian | C |
| 24 | DingnanXian | D | 140 | ShanghangXian | A |
| 25 | DongshanXian | EX | 141 | ShangraoXian | A |
| 26 | DongtouXian | EX | 142 | ShangyouXian | D |
| 27 | DongxiangXian | EX | 143 | ShangyuShi | EX |
| 28 | DongyangShi | EX | 144 | ShaowuShi | A |
| 29 | DuchangXian | EX | 145 | ShaoxingShiShixiaqu | EX |
| 30 | FengchengShi | C | 146 | ShaoxingXian | EX |
| 31 | FenghuaShi | EX | 147 | ShaXian | D |
| 32 | FengxinXian | B | 148 | ShengsiXian | EX |
| 33 | FenyiXian | C | 149 | ShengzhouShi | C |
| 34 | FuanShi | EX | 150 | ShichengXian | A |
| 35 | FudingShi | EX | 151 | ShishiShi | EX |
| 36 | FuliangXian | C | 152 | ShouningXian | D |
| 37 | FuqingShi | EX | 153 | ShunchangXian | D |
| 38 | FuyangShi | C | 154 | SongxiXian | D |
| 39 | FuzhouShiShixiaqu | EX | 155 | SongyangXian | A |
| 40 | GanXian | D | 156 | SuichangXian | A |
| 41 | GanzhouShiShixiaqu | C | 157 | SuichuanXian | D |
| 42 | GaoanShi | C | 158 | TaiheXian | C |
| 43 | GuangchangXian | A | 159 | TainingXian | A |
| 44 | GuangfengXian | A | 160 | TaishunXian | C |
| 45 | GuangzeXian | A | 161 | TaizhouShiShixiaqu | EX |
| 46 | GuixiShi | C | 162 | TiantaiXian | EX |
| 47 | GutianXian | D | 163 | TongguXian | A |
| 48 | HainingShi | EX | 164 | TongluXian | C |
| 49 | HaiyanXian | EX | 165 | TongxiangShi | EX |
| 50 | HangzhouShiShixiaqu | EX | 166 | WananXian | C |
| 51 | HengfengXian | C | 167 | WannianXian | EX |
| 52 | HuaanXian | D | 168 | WanzaiXian | C |
| 53 | HuianXian | EX | 169 | WenchengXian | D |
| 54 | HuichangXian | A | 170 | WenlingShi | EX |
| 55 | HukouXian | EX | 171 | WenzhouShiShixiaqu | EX |
| 56 | HuzhouShiShixiaqu | EX | 172 | WuningXian | C |
| 57 | JiachengQu | C | 173 | WupingXian | A |
| 58 | JiandeShi | D | 174 | WuyishanShi | A |
| 59 | JiangleXian | A | 175 | WuyiXian | A |
| 60 | JiangshanShi | A | 176 | WuyuanXian | D |
| 61 | JianningXian | A | 177 | XiajiangXian | EX |
| 62 | JianouXian | D | 178 | XiamenShiShixiaqu | EX |
| 63 | JianXian | C | 179 | XiangshanXian | EX |
| 64 | JianyangShi | D | 180 | XianjuXian | C |
| 65 | JiashanXian | EX | 181 | XianyouXian | EX |
| 66 | JiaxingShiShixiaqu | EX | 182 | XiaoshanQu | EX |
| 67 | JindongQu | A | 183 | XiapuXian | EX |
| 68 | JinganXian | A | 184 | XinchangXian | EX |
| 69 | JingdezhenShiShixiaqu | C | 185 | XinfengXian | D |
| 70 | JinggangshanShi | A | 186 | XinganXian | C |
| 71 | JingningShezuZizhixian | A | 187 | XingguoXian | D |
| 72 | JinhuaShiShixiaqu | C | 188 | XingziXian | C |
| 73 | JinjiangShi | EX | 189 | XinjianXian | EX |
| 74 | JinmenXian | EX | 190 | XinyuShiShixiaqu | EX |
| 75 | JinxianXian | EX | 191 | XinzhouQu | EX |
| 76 | JinxiXian | C | 192 | XiushuiXian | C |
| 77 | JinyunXian | D | 193 | XunwuXian | A |
| 78 | JishuiXian | C | 194 | YanshanXian | A |
| 79 | JiujiangShiShixiaqu | C | 195 | YifengXian | C |
| 80 | JiujiangXian | EX | 196 | YihuangXian | A |
| 81 | JizhouQu | EX | 197 | YingtanShiShixiaqu | EX |
| 82 | KaihuaXian | A | 198 | YinXian | EX |
| 83 | LanxiShi | C | 199 | YiwuShi | C |
| 84 | LeanXian | C | 200 | YiyangXian | C |
| 85 | LepingShi | EX | 201 | YonganShi | A |
| 86 | LianchengXian | A | 202 | YongchunXian | EX |
| 87 | LianduQu | A | 203 | YongdingXian | D |
| 88 | LianhuaXian | C | 204 | YongfengXian | C |
| 89 | LianjiangXian | EX | 205 | YongjiaXian | C |
| 90 | LichuanXian | A | 206 | YongkangShi | C |
| 91 | LinanShi | C | 207 | YongtaiXian | C |
| 92 | LinchuanQu | C | 208 | YongxinXian | C |
| 93 | LinhaiShi | EX | 209 | YongxiuXian | C |
| 94 | LonghaiShi | EX | 210 | YouxiXian | D |
| 95 | LongnanXian | C | 211 | YuanzhouQu | C |
| 96 | LongquanShi | A | 212 | YuduXian | D |
| 97 | LongyanShiShixiaqu | A | 213 | YueqingShi | C |
| 98 | LongyouXian | D | 214 | YuganXian | EX |
| 99 | LuoyuanXian | EX | 215 | YuhangQu | EX |
| 100 | MingxiXian | A | 216 | YuhuanXian | EX |
| 101 | MinhouXian | C | 217 | YujiangXian | EX |
| 102 | MinqingXian | C | 218 | YunheXian | A |
| 103 | NananShi | EX | 219 | YunxiaoXian | C |
| 104 | NanchangShiShixiaqu | EX | 220 | YushanXian | D |
| 105 | NanchangXian | EX | 221 | YuyaoShi | EX |
| 106 | NanchengXian | D | 222 | ZhangpingShi | A |
| 107 | NanfengXian | D | 223 | ZhangpuXian | C |
| 108 | NanjingXian | C | 224 | ZhangshuShi | C |
| 109 | NankangShi | C | 225 | ZhangzhouShiShixiaqu | EX |
| 110 | NanpingShiShixiaqu | D | 226 | ZhaoanXian | C |
| 111 | NingboShiShixiaqu | EX | 227 | ZhengheXian | D |
| 112 | NingduXian | A | 228 | ZherongXian | EX |
| 113 | NinghaiXian | EX | 229 | ZhouningXian | D |
| 114 | NinghuaXian | A | 230 | ZhoushanShiShixiaqu | EX |
| 115 | PananXian | C | 231 | ZhujiShi | C |
| 116 | PengzeXian | C | 232 | ZixiXian | A |

Note: Quadrant is associated to Figure 4 in the article. A means the priority prefecture (19.83%); B & D means the important prefecture (15.52%); C means the normal prefecture (29.74%); EX means the prefecture excluded, because the cell of the potential habitat in the 2000s is less than 20 cells, 34.91% was excluded.
