# Supplementary material for: Historical data for conservation: reconstructing range changes of Chinese pangolin (Manis pentadactyla) in eastern China (1970–2016)
Source: Proc Biol Sci. 2018 Aug 22;285(1885):20181084. doi: 10.1098/rspb.2018.1084 (PMC6125891; doi:10.1098/rspb.2018.1084)
Supplement: Appendix IV Influence from climate change [file rspb20181084supp4.docx]

*Historical data for conservation: reconstructing range changes of Chinese pangolin (Manis pentadactyla) in eastern China (1970-2016)*

Li Yang, Minhao Chen, Daniel W.S. Challender, Carly Waterman, Chao Zhang, Zhaomin Huo, Hongwei Liu, Xiaofeng Luan

**Appendix IV Influence from climate change**

Climate change has been demonstrated to have a significant impact on species’ distributions and diversity patterns [1-3]. It is reasonable to assume that the range of Chinese pangolin may decrease due to climate change. Here we assume that human influence has remained stable for decades. In this case, we combined the climate layers (including MAT, MAP, NFFD, Eref) in the 2000s with the human influence layers (the Forestry and Urban Land) in the 1970s, and climate layer from 2000s and the human influence layers in the 1980s. We selected two periods including the 1970s and the 1980s, because range change under climate change may take decades. Then, we created two assumption layers for the 2000s including 8 variables (4 climate layers, 2 human influence layers, and 2 topography layers).

Following the method (species distribution modelling) from the article, we project the potential habitat in the 2000s with two assumption layers and the occurrence points for two periods (Fig S1). Then, we compared the “true” result with two assumptions in the 2000s. Here is the hypothesis: if the climate change is negative factor, the assumptions in the 2000s will smaller than the true range.


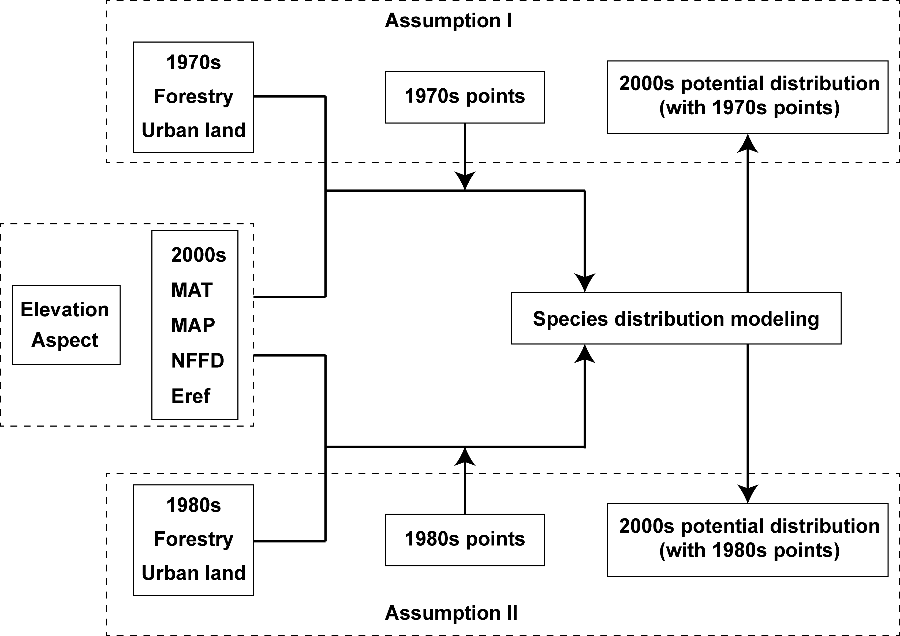


Figure S1 brief method for the test under two assumptions

The test results show that the potential distribution for two assumptions tends to be larger in the 2000s compared with the “true” result: 257,006 km^2^ for assumption I, 83.5% increase in the range in the 1970s; 214,198 km^2^ for assumption II, 105.8% increase in the range in the 1980s (Fig S2). Due to dispersal limitation, and reproductive parameters, the Chinese pangolin may not colonize all potential habitat patches [4-6]. Moreover, the potential distribution of these assumptions tends to larger compared with the results of 1970s (64.82% increase) or 1980s (105.78% increase). Indeed, the increasing under the two assumptions cannot prove that the climate change is a positive factor for Chinese pangolin. In fact, this test provides evidence to support that climate condition can be neutral factor, and may be positive in some cases. In other words, human interference could be responsible for the range decrease of Chinese pangolins.


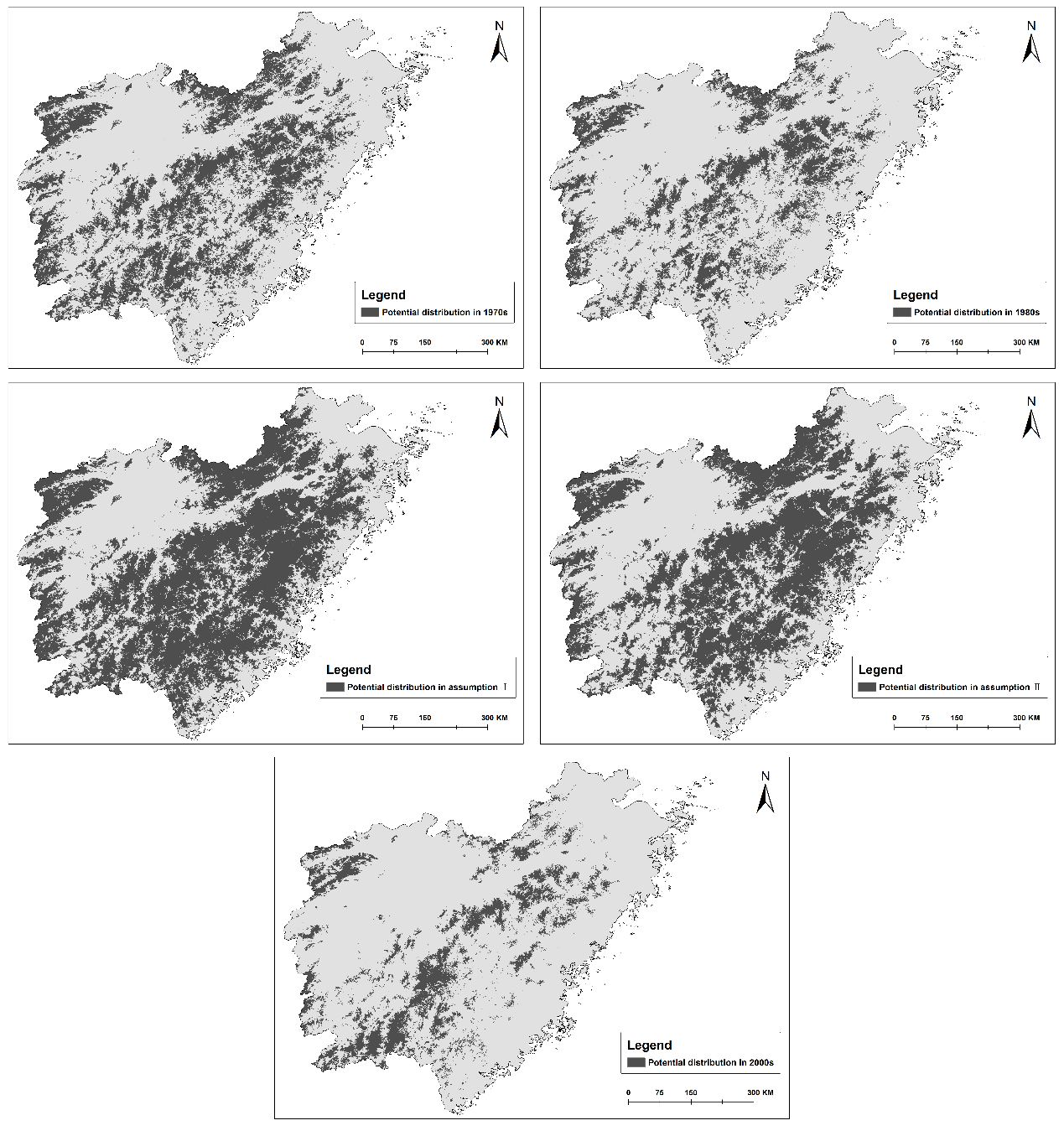


Figure S2 Potential distribution in two assumptions and the result from the 2000s

**Reference**

[1] Urban, M.C., Zarnetske, P.L. & Skelly, D.K. 2013 Moving forward: dispersal and species interactions determine biotic responses to climate change. *Ann N Y Acad Sci* **1297**, 44-60. (doi:10.1111/nyas.12184).

[2] Luo, Z., Jiang, Z. & Tang, S. 2015 Impacts of climate change on distributions and diversity of ungulates on the Tibetan Plateau. *Ecological Applications* **25**, 24-38. (doi:abs/10.1890/13-1499.1).

[3] Pacifici, M., Foden, W.B., Visconti, P., Watson, J.E.M., Butchart, S.H.M., Kovacs, K.M., Scheffers, B.R., Hole, D.G., Martin, T.G., Akçakaya, H.R., et al. 2015 Assessing species vulnerability to climate change. *Nature Climate Change* **5**, 215-224. (doi:10.1038/nclimate2448).

[4] Wu, S. 1998 Notes on a newborn Chinese Pangolin (*Manis Pentadactyla Aurita*). *Journal of Qinghai Normal University (Natural Science)* **1**, 40-42. (doi:10.16229 /j.cnki.issn1001-7542.1998.01.010).

[5] Wu, S.B., LIU, N., Zhang, Y. & MA, G.Z. 2010 Assessment Of Threatened Status Of Chinese Pangolin (*Manis Pentadactyla*). *CHINESE JOURNAL OF APPLIED & ENVIRONMENTAL BIOLOGY* **10**, 456-461. (doi:10.3321/j.issn:1006-687X.2004.04.014).

[6] Zhang, F.H., Wu, S.B., Yang, L., Zhang, L., Sun, R.Y. & Li, S.S. 2015 Reproductive parameters of the Sunda pangolin, *Manis javanica*. *Folia Zool* **64**, 129-135.
